# Efficient sequential harvesting of solar light by heterogeneous hollow shells with hierarchical pores

**DOI:** 10.1093/nsr/nwaa059

**Published:** 2020-04-08

**Authors:** Yanze Wei, Jiawei Wan, Nailiang Yang, Yu Yang, Yanwen Ma, Songcan Wang, Jiangyan Wang, Ranbo Yu, Lin Gu, Lianhui Wang, Lianzhou Wang, Wei Huang, Dan Wang

**Affiliations:** State Key Laboratory of Biochemical Engineering, Institute of Process Engineering, Chinese Academy of Sciences, Beijing 100190, China; Department of Physical Chemistry, School of Metallurgical and Ecological Engineering, University of Science and Technology Beijing, Beijing 100083, China; State Key Laboratory of Biochemical Engineering, Institute of Process Engineering, Chinese Academy of Sciences, Beijing 100190, China; State Key Laboratory of Biochemical Engineering, Institute of Process Engineering, Chinese Academy of Sciences, Beijing 100190, China; University of Chinese Academy of Sciences, Chinese Academy of Sciences, Beijing 100049, China; State Key Laboratory of Biochemical Engineering, Institute of Process Engineering, Chinese Academy of Sciences, Beijing 100190, China; School of Materials Science and Engineering, Nanjing University of Posts and Telecommunications, Nanjing 210046, China; School of Chemical Engineering and Australian Institute for Bioengineering and Nanotechnology, The University of Queensland, St Lucia 4072, Australia; State Key Laboratory of Biochemical Engineering, Institute of Process Engineering, Chinese Academy of Sciences, Beijing 100190, China; Department of Physical Chemistry, School of Metallurgical and Ecological Engineering, University of Science and Technology Beijing, Beijing 100083, China; Institute of Physics, Chinese Academy of Sciences, Beijing 100190, China; School of Materials Science and Engineering, Nanjing University of Posts and Telecommunications, Nanjing 210046, China; School of Chemical Engineering and Australian Institute for Bioengineering and Nanotechnology, The University of Queensland, St Lucia 4072, Australia; School of Materials Science and Engineering, Nanjing University of Posts and Telecommunications, Nanjing 210046, China; State Key Laboratory of Biochemical Engineering, Institute of Process Engineering, Chinese Academy of Sciences, Beijing 100190, China; University of Chinese Academy of Sciences, Chinese Academy of Sciences, Beijing 100049, China

**Keywords:** light harvesting, hollow structures, multi-shelled, solar energy conversion

## Abstract

In nature, sequential harvesting of light widely exists in the old life entity, i.e. cyanobacteria, to maximize the light absorption and enhance the photosynthesis efficiency. Inspired by nature, we propose a brand new concept of temporally-spatially sequential harvesting of light in one single particle, which has purpose-designed heterogeneous hollow multi-shelled structures (HoMSs) with porous shells composed of nanoparticle subunits. Structurally, HoMSs consist of different band-gap materials outside-in, thus realizing the efficient harvesting of light with different wavelengths. Moreover, introducing oxygen vacancies into each nanoparticle subunit can also enhance the light absorption. With the benefit of sequential harvesting of light in HoMSs, the quantum efficiency at wavelength of 400 nm is enhanced by six times compared with the corresponding nanoparticles. Impressively, using these aforementioned materials as photocatalysts, highly efficient photocatalytic water splitting is realized, which cannot be achieved by using the nanoparticle counterparts. This new concept of temporally-spatially sequential harvesting of solar light paves the way for solving the ever-growing energy demand.

## Teaser

Mimicking the antenna system of cyanobacteria, the sequential harvesting of solar light is achieved by temporal-spatial ordered hollow multishell structures for efficient solar energy conversion.

## INTRODUCTION

Solar energy conversion is an ideal path for addressing the global energy and environmental crisis [1–3]. Among the various steps of the solar energy conversion process, sunlight harvesting is the primary step, which determines the energy conversion efficiency [4–8]. Natural photosynthesis is the ultimate model for solar-to-chemical conversion, which provides the solution to enhancing sunlight harvesting efficiency [9–12]. In the antenna system of cyanobacteria, which represents the oldest life entity on our earth and accumulates large amounts of oxygen for oxygenic life, different antenna pigments are loaded in a certain order to realize the sequential collection of light energy (from the outside to the inside: phycoerythrin (570 nm), phycocyamin (630 nm), allophycocyanin (650 nm), chlorophyll α (670–678 nm)), thus reaching the maximum light absorption (Supplementary Fig. 1). As a result, this structure ensures a fast and precise route to driving the redox reaction and completing the energy conversion process [13–15].

As reported, nature-inspired materials have been long desired by human society, as they generally possess unexpected properties and show great advantages in many fields [16–20]. To mimic the sequential light harvesting in the antenna system of cyanobacteria and realize efficient light utilization, the desired photosynthetic system should meet the following requirements from structure to function: (1) multi-shelled membrane system to confine the photosynthetic process; (2) sequential harvesting of light in antenna system (absorb the shorter wavelength outside, and absorb the longer wavelength inside) to improve light absorption efficiency; (3) porous shells on the membrane for mass transfer; and (4) short route for fast charge transport [21–23].

Herein, inspired by the ingenious natural photosynthesis system, three-dimensional (3D) hollow multi-shelled structures (HoMSs) with porous shells composed of zero-dimensional (0D) nanoparticle subunits are constructed to establish the artificial photosynthesis system [24–26], which meets the aforementioned requirements as: (1) the size and the multiple shell structure of 3D HoMSs are proper to perform as nano-micro reactors; (2) most importantly, the sequential harvesting of sunlight in HoMSs is realized by introducing various materials with different band gaps into different shells. The temporal-spatial ordering of heterogeneous shells enables the weakly penetrable short-wavelength light and strongly penetrable long-wavelength light to be preferentially absorbed by the outer and inner part of HoMSs, respectively, thus greatly improving light absorption efficiency and ensuring dispersed excitation events on distinct shells. Furthermore, the sequential light harvesting can also be realized by introducing oxygen vacancies onto the surface of each 0D subunit, thus enhancing light utilization; (3) the porous shells are similar to the membrane system, which facilitates mass transfer; and (4) the thin shell of HoMSs could shorten the diffusion paths of photo-excited carriers [27–30]. Owing to the biomimetic structure and functions analogous to the antenna system of cyanobacteria, the 3D heterogeneous HoMSs system is highly expected to show promising potential in realizing efficient artificial photoreaction [31].

**Figure 1.**
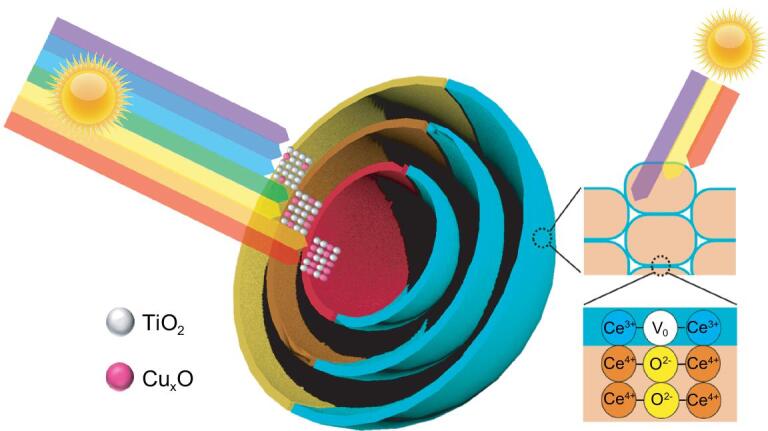
Illustration of two designed heterogeneous HoMSs for efficient sequential harvesting of solar light. The left part represents TCHoMSs, while the right part represents CFHoMSs. V_O_ indicates oxygen vacancy. The left part of the scheme shows an increased molar ratio of Ti/Cu along outside-in TiO_2_-Cu_x_O shells, which enables sequential harvesting of weakly penetrable short-wavelength light- by the outer part and strongly penetrable long-wavelength light- by the inner part. The different colors of the multiple shells indicate their different compositions. The right part of the scheme shows that abundant oxygen vacancies exist on the surface of CeO_2_-CeFeO_3_ nanoparticle subunits, thus realizing sequential light- harvesting from the surface to the inside of each nanoparticle subunit.

As Fig. 1 shows, TiO_2_-Cu_x_O hollow multi-shelled structures (TCHoMSs) and CeO_2_-CeFeO_3_ hollow multi-shelled structures (CFHoMSs) with different compositional nanoparticle subunits and shells were fabricated to realize sequential harvesting of solar light. For TCHoMSs, the outer part is mainly composed of broad-band-gap TiO_2_ semiconductors (Ti/Cu = 15:1) that can absorb weakly penetrable short-wavelength light, while the inner part consists of increased ratio of narrow-band-gap Cu_x_O semiconductors (Ti/Cu = 6:1) that can absorb strongly penetrable long-wavelength light. In addition, sequential light harvesting of each 0D subunit of the triple-shelled CeO_2_-CeFeO_3_ hollow structures (3S-CFHoMSs) was realized by introducing oxygen vacancies into the surface of each nanoparticle subunit. To experimentally prove the benefits of our newly proposed concept, photocatalytic water splitting was chosen as a model reaction, using purpose-designed heterogeneous HoMSs as photocatalysts. As a result, quadruple-shelled TiO_2_-Cu_x_O hollow structures (4S-TCHoMSs) presented significantly enhanced hydrogen evolution activity and stability (2490 μmol/h, 90 h), compared to TiO_2_-Cu_x_O nanoparticles or reversed TiO_2_-Cu_x_O heterostructures. Excellent oxygen evolution rate (452 μmol/h) was also achieved by 3S-CFHoMSs, which greatly benefited from the surface oxygen vacancies and heterojunctions on each subunit. Owing to the enhanced light absorption, the overall water splitting based on particle suspension system was achieved by combining 4S-TCHoMSs and 3S-CFHoMSs with the aid of redox shuttle.

### Synthesis and characterization of two heterogeneous HoMSs

The accurate synthesis of HoMSs remains a challenge, especially for obtaining distinct compositions within different shells to realize sequential light- harvesting [32]. Herein, TiO_2_-Cu_x_O hollow multi-shelled structures with tunable shell number (single-, double-, triple- and quadruple-shelled) and different inner/outer shell composition were synthesized through a sequential template method by accurately controlling the cation absorption process of carbon microsphere (CMS) templates. By adjusting ion concentrations, ion radius and ion charges, the distribution and amount of adsorbed precursors in CMS templates can be well controlled, thus the shell composition can be purposely tuned [33–36]. On one hand, the Ti precursor concentration is much higher than Cu precursor concentration, leading to a higher overall ratio of Ti in the TiO_2_-Cu_x_O products. On the other hand, [Cu(H_2_O)_4_]^2+^ cation has a smaller hydrated ionic radius and is more positively charged than Ti coordination cations [Ti(OH)_n_(H_2_O)_6-n_]^(4-n)+^ (n = 2, 3), thus [Cu(H_2_O)_4_]^2+^ cations can be more easily adsorbed into the center of CMS, resulting in a molar ratio gradient of Ti/Cu [37,38]. After removing the CMS templates through a combustion process, TCHoMSs with different shell compositions are fabricated. Besides, increase of adsorption temperature and duration can improve the absorption depth and amount of precursors, thus increasing the shell number of hollow structures.

**Figure 2.**
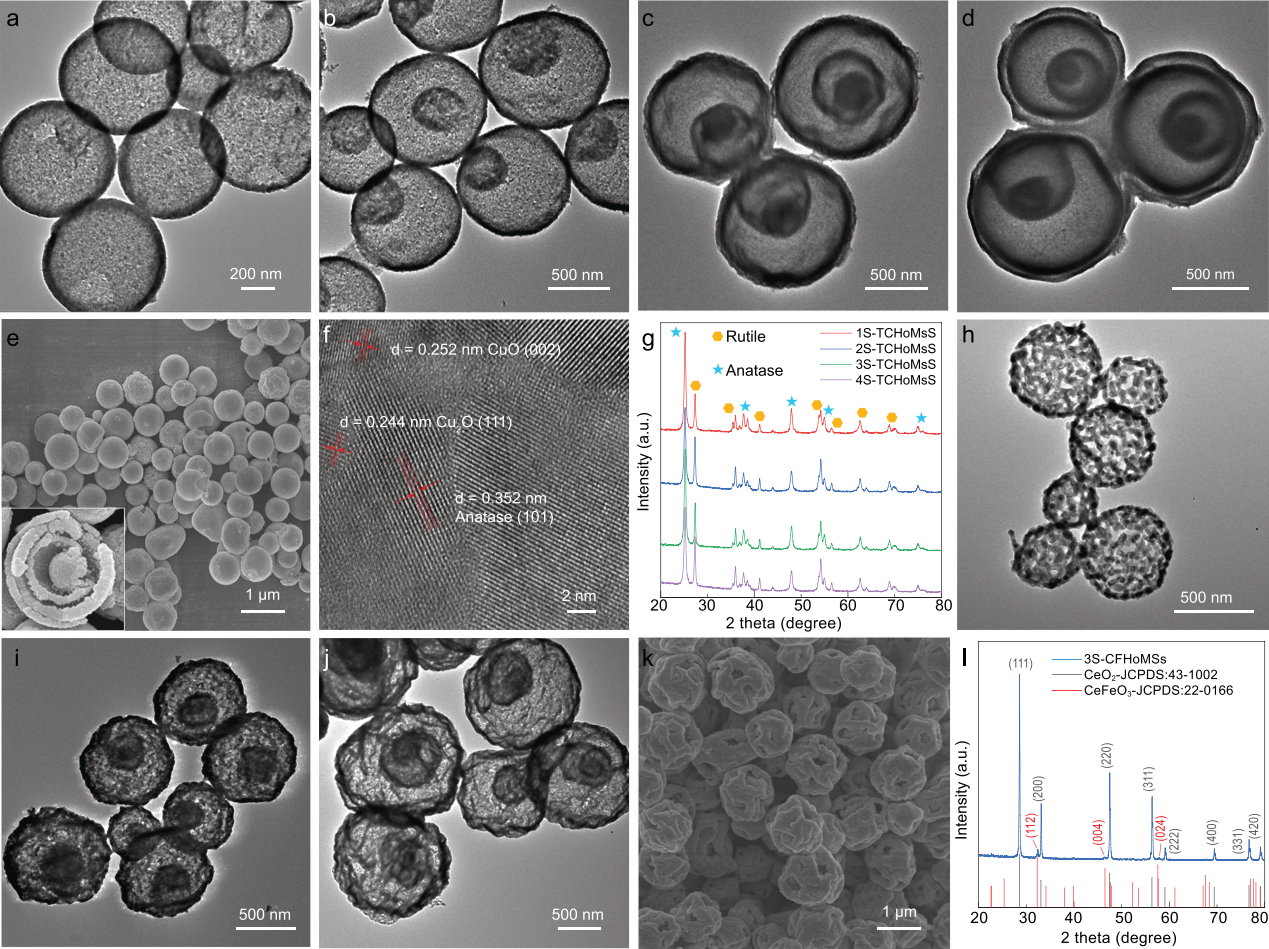
Morphological and structural analysis of as-prepared HoMSs. (a–d) TEM images and (e) SEM image of TCHoMSs: (a) single-shelled (1S-), (b) double-shelled (2S-), (c) triple-shelled (3S-) and (d, e) quadruple-shelled (4S-) TCHoMSs (inset is the high-magnification SEM image of one typical microsphere); (f) HRTEM image of TCHoMSs; (g) XRD patterns of TCHoMS samples; (h–j) TEM images of CeO_2_-CeFeO_3_ CFHoMSs: (h) single-shelled (1S-), (i) double-shelled (2S-) and (j) triple-shelled (3S-) CFHoMSs; (k) SEM image of 3S-CFHoMSs; (l) XRD pattern of 3S-CFHoMSs.

Scanning electron microscopy (SEM) images illustrate the uniform spherical morphology of TCHoMSs with a size around 600–800 nm (Fig. 2a–d), of which the inner hierarchical structure is clearly shown in the inserted image (Fig. 2e).

Transmission electron microscopy (TEM) images show that the shells are composed of tiny crystal grains (size: ∼17.6 nm, similar to ∼15.8 nm derived from X-ray diffraction (XRD) calculation), and the thicknesses of shells range from 34 to 62 nm (Supplementary Fig. 2). Besides, the lattice spacings of 0.352 and 0.325 nm correspond to anatase (101) plane and rutile (110) plane, respectively, indicating that different TiO_2_ phases exist in the sample, while the observed lattice spacings of 0.252 nm for CuO (002) plane and 0.244 nm for Cu_2_O (111) plane prove that the Cu_x_O phases are attached tightly to the TiO_2_ particles (Fig. 2f). As shown in XRD patterns (Fig. 2g), the main peaks of samples with different numbers of shells are indexed to TiO_2_ (anatase: JCPDS card no. 21–1272 and rutile: JCPDS card no. 21–1276). Although the peaks of copper oxides are not observed in the XRD patterns, which is possibly due to the low concentration of Cu_x_O in the material being below the detection limit of XRD, the Cu 2p peak located at 935 eV in X-ray photoelectron spectroscopy (XPS) spectrum of TCHoMSs confirmed the existence of Cu element (Supplementary Fig. 3a).

Further information from high-resolution XPS spectra of Cu 2p orbital indicates that Cu element is mainly composed of Cu (I) and Cu (II) with a molar ratio of 6:5 (Supplementary Fig. 3b). Similar Cu (I) and Cu (II) ratio was observed for the TiO_2_-Cu_x_O nanoparticles (Supplementary Fig. 3c). Comparing the laser Raman spectra of pure TiO_2_ and 4S-TCHoMSs, no peak shifts were observed, which confirmed that no Cu doping was detected (Supplementary Fig. 3d).

Furthermore, CeO_2_-CeFeO_3_ HoMSs were prepared through a similar sequential template method to realize the sequential light harvesting of each 0D nanoparticle subunit of the 3D HoMSs. After a specific calcination process, single-, double- and triple-shelled CeO_2_-CeFeO_3_ hollow structures with big pores on the surface are obtained, as shown by TEM images (Fig. 2h–j). SEM image illustrates the porous surface more clearly, where small crystals sintered together to form the shells as well as create big holes in the shells (Fig. 2k). XRD pattern of the triple-shelled sample shows that the main peaks are indexed to CeO_2_ phase (JCPDS card no. 43–1002) and CeFeO_3_ phase (JCPDS card no. 22–0166) (Fig. 2l).

### Sequential absorption of light in as-prepared HoMSs

**Figure 3.**
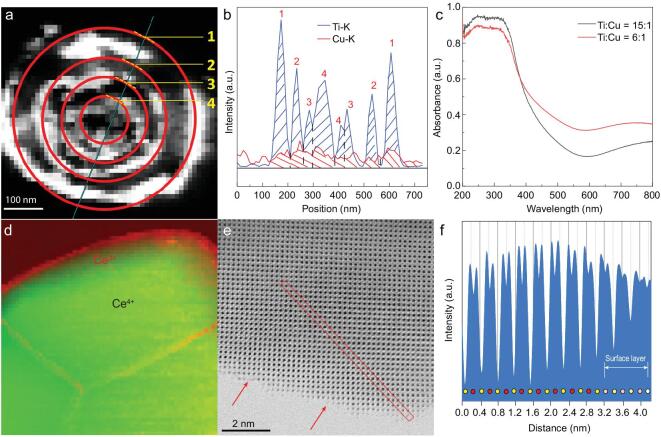
Structural characterization and sequential light absorption of TCHoMSs and CFHoMSs. (a) Dark-field TEM image of a slice of 4S-TCHoMSs; (b) EDS line scanning along the cyan line in (a), showing the Ti/Cu ratios in different shells; (c) absorption spectrum of TiO_2_-Cu_x_O single-shelled hollow structures with Ti/Cu molar ratio of 15:1 and 6:1; (d) EELS mapping of Ce^3+^ and Ce^4+^, showing their distribution in the crystals (green: Ce^4+^; red: Ce^3+^); (e) the spherical aberration-corrected STEM image of 3S-CFHoMSs (the single crystal surface, with red arrows indicating the atom steps); (f) the corresponding line profile of the area in the red rectangle in (e) with the direction from the upper left to the bottom right.

To qualitatively study the relative molar ratio of Ti/Cu in different shells, 4S-TCHoMSs slices with a thickness around 40 nm were obtained with the aid of resin embedding agent (Fig. 3a). The molar ratio of Ti/Cu is estimated by energy dispersive X-ray spectroscopy (EDS). The Ti/Cu ratio in the outmost shell is around 15:1, while the inner shells have an increased Cu content, which has a Ti/Cu ratio of approximately 6:1 (Fig. 3b). To further analyse the absorption spectrum of different layers of 4S-TCHoMSs, TiO_2_-Cu_x_O single-shelled hollow structures with two different Ti/Cu molar ratios of 15:1 and 6:1 are synthesized through the similar sequential template approach. The Ti/Cu ratio is further confirmed by inductively coupled-plasma mass spectrometry (ICP-MS) (Supplementary Tables 1 and 2). As illustrated in Fig. 3c, the hollow spheres with Ti/Cu ratio of 15:1 show higher ultraviolet (UV) light absorbance due to the higher portion of TiO_2_ composition, indicating that UV light can be better absorbed by the outmost shell of 4S-TCHoMSs. In comparison, a conspicuous increase of absorption in the spectrum of 400–600 nm is achieved for the hollow structures with Ti/Cu ratio of 6:1. The result indicates that inner shells of 4S-TCHoMSs have better response to visible light (Supplementary Fig. 4). As it is acknowledged, diffraction ability of light is enhanced with the increase of wavelength [39,40]. Visible light is more likely to diffract through the porous shell and be absorbed by the inner shells, while the short-wavelength UV light is more easily directly absorbed by the outer shells. As a result, by deliberately developing a ratio gradient of Ti/Cu along the shells, sequential absorption of sunlight is achieved in a sequence from UV light to visible light.

**Figure 4.**
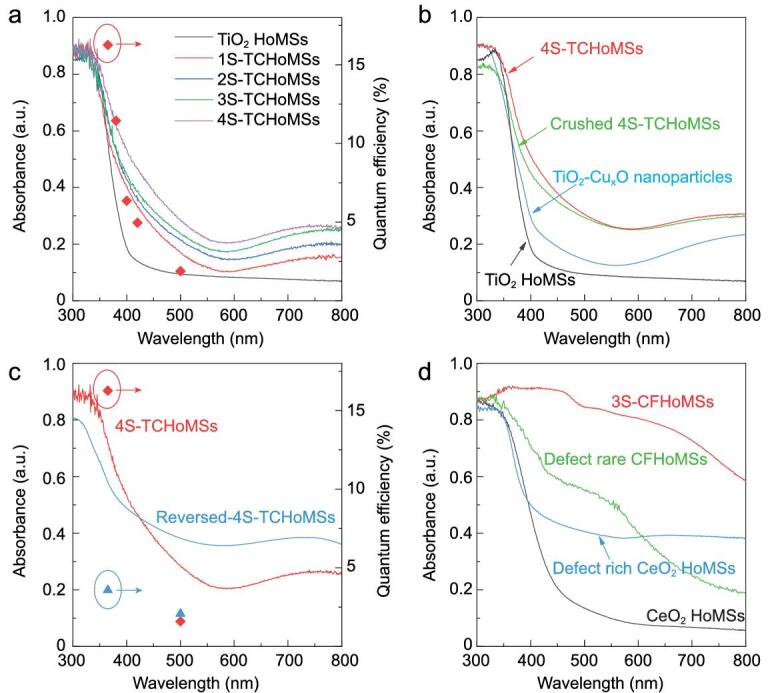
Sequential light absorption properties of HoMSs. (a) UV-Vis absorption curves of TCHoMSs with different shell numbers and apparent quantum efficiency (red diamonds) of 4S-TCHoMSs at different wavelengths; (b) UV-Vis absorption curves of 4S-TCHoMSs TiO_2_-Cu_x_O nanoparticles, crushed 4S-TCHoMSs and TiO_2_ HoMSs; (c) UV-Vis absorption curves and apparent quantum efficiency of 4S-TCHoMSs (red diamonds) and reversed 4S-TCHoMSs (blue triangles); (d) UV-Vis absorption curves of 3S-CFHoMSs, CeO_2_ HoMSs and the corresponding samples with surface defect control.

In addition to the chemical composition of shells, the light-harvesting ability is also related to the number of shells and the unique hollow structure. The ultraviolet-visible (UV-Vis) absorption curves demonstrate that the entire light absorption increases with the increase of shell number (Fig. 4a). Notably, with the introduction of space sequential light-harvesting structures, the 4S-TCHoMSs exhibited the highest absorption in visible light region. Moreover, to quantify the spectral distribution of the photocatalytic activity of 4S-TCHoMSs, the apparent quantum efficiency (AQE) for hydrogen evolution reaction (HER) was measured at single wavelength of 365, 400, 420 and 500 nm. The AQE decreases obviously with increasing wavelength, and the detectable efficiency calculated at 500 nm indicates that the existence of Cu_x_O enables the UV responsive TiO_2_ to carry on HER reaction within visible light region. The enhancement in light-harvesting ability could be further observed by comparing the UV-Vis spectra of TCHoMSs with those of TiO_2_-Cu_x_O nanoparticles (TCNPs), TiO_2_ HoMSs and the corresponding crushed 4S-HoMSs (Fig. 4b). The collapse of the highly ordered structures greatly influenced light scattering and reduced light absorption in a broad wavelength (Supplementary Fig. 5). Interestingly, although the inner shells had more Cu_x_O content exposed after the HoMSs were crushed, the light absorption in visible light region still showed a slight decrease. In addition, 4S-TCHoMSs with reversed TiO_2_ and Cu_x_O compositions were synthesized to demonstrate the importance of light-harvesting sequence (Supplementary Fig. 6). Owing to higher Cu_x_O content in the outer shells, the reversed 4S-TCHoMSs showed enhanced visible light absorption, but the UV absorption was greatly hindered (Fig. 4c), which was also conspicuously reflected by the different AQE of HER (Fig. 4c).

Additionally, we intentionally designed CFHoMSs, in which oxygen vacancies exist on the surface of each 0D nanoparticle subunit for efficient harvesting of solar light. The surface of a single crystal is highlighted in the high-resolution spherical aberration-corrected scanning transmission electron microscopy (STEM) image (Fig. 3e). Atomic steps are visibly presented and marked with red arrows, indicating the atom absence at the surface. The inter-atomic distance obtained by line profiles along the red section shows that the oxygen vacancies are mainly distributed at the surface area of the grains [41]. Besides, electron energy loss spectroscopy (EELS) demonstrates that Ce^3+^ mainly concentrates at the outer surface with a thickness around 3.3 nm, indicating that surface defects locate at the edge of the crystals and grain boundaries [42] (Fig. 3d, red edge), while the body part of the crystalline grain is CeO_2_ (Fig. 3d, green part). The increased Ce^3+^ portion and oxygen vacancies are also confirmed by the high-resolution XPS spectra of Ce 3d and O 1s orbits. After the treatment in reductive atmosphere, the peaks attributed to Ce^3+^ and the chemisorbed peroxide oxygen (O_2_^2−^), which acts as the active oxygen, increased remarkably [43] (Supplementary Fig. 7a and b). As described in previous experimental and theoretical studies, these oxygen vacancies function as active centers for gas evolution reaction, and water molecules are inclined to be adsorbed by the Ce^3+^ ions or oxygen vacancies, which reduce the energy barrier of water oxidation [43,44]. Owing to the surface oxygen vacancies and CeFeO_3_ phase, these CFHoMSs exhibited an enhanced light absorption with a broader absorption edge of visible light as illustrated in Fig. 4b. The creation of surface oxygen vacancies and combination with CeFeO_3_ significantly increased the light absorption of each subunit of CFHoMSs. The effect of surface oxygen vacancies in light absorption could be testified by comparing the UV-Vis spectra of CFHoMSs and CFHoMSs after oxygen treatment at 800°C. 3S-CFHoMSs exhibited the highest light absorption both in the UV and visible region, much stronger than CeO_2_ HoMSs with surface oxygen vacancies and CeO_2_-CeFeO_3_ without surface oxygen vacancies. As expected, the 3S-CFHoMS sample, which has the highest shell number, showed the best light absorption ability as well as the lowest photoluminescence intensity (Supplementary Fig. 7c and d).

### Water splitting performance

**Figure 5.**
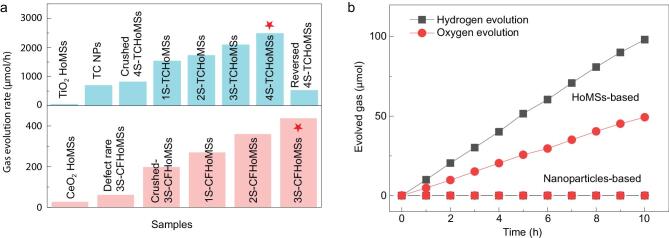
Water splitting performance of TCHoMSs and CFHoMSs. (a) Hydrogen evolution activity and oxygen evolution activity of TCHoMSs and CFHoMSs related samples under 300 W Xe lamp irradiation at 281 K; (b) the overall water splitting performance of 4S-TCHoMSs and CFHoMSs under 300 W Xe lamp irradiation at 281 K.

To prove the concept of sequential light harvesting, we chose photocatalytic water splitting as a model reaction. TCHoMSs display excellent hydrogen evolution rate under 300 W Xe lamp irradiation in the solution of methanol and deionized water. The linear hydrogen evolution curves of TCHoMSs (Supplementary Fig. 8a)

indicate the stable reaction within six hours. As expected, all the TCHoMSs showed better HER properties than the TCNPs with the same content. As shell number increases, the enhanced hydrogen evolution performance of TCHoMSs further confirmed the benefit from the sequential light-harvesting design [45]. Impressively, 4S-TCHoMSs exhibited the best performance with a hydrogen evolution rate of 2490 μmol/h (Fig. 5a). Importantly, more than 95% of the original activity after 16 cycles (96 hours) remained for 4S-TCHoMSs, which benefited from the inhibited photocorrosion due to the suppressed charge carrier accumulation [46], and the impressive structural stability of the HoMSs attributed to the multiple shells supporting each other and the exterior shell protecting the interior shells (Supplementary Fig. 8c).

Furthermore, to evaluate practical application feasibility of the photocatalyst, the performance of the as-synthesized samples under simulated sunlight with AM 1.5 G illumination is also measured (Supplementary Fig. 9). Impressively, owing to the introduction of Cu_x_O and a fine sequential absorption structure, the 4S-TCHoMSs exhibited the best performance (320 μmol/h) among the TCHoMSs and considerably surpassed both TCNPs and TiO_2_ HoMSs. The hydrogen evolution activity and stability of as-prepared 4S-TCHoMSs are the best among the reported TiO_2_-based materials (Supplementary Table 4). The band structure of TCHoMSs was confirmed by UV-Vis spectra and Mott-shcottky measurements, as illustrated in Supplementary Fig. 10. In addition, the electron transport paths between TiO_2_ and Cu_x_O were revealed by photocurrent responses under different incident light wavelength (Supplementary Fig. 11) [47].

The importance of sequential light harvesting on the selected 4S-TCHoMSs in the application of photocatalytic HER was also verified by TCHoMSs with reversed TiO_2_ and Cu_x_O compositions. Owing to the increased content of Cu, although slight increase of visible light driven photocatalytic HER performance was observed, the performance under Xe lamp irradiation decreased to almost one-fifth of the 4S-TCHoMSs (Supplementary Fig. 6c). The different HER performance verified that sequential light harvesting is significant for light utilization in HoMSs, as nature shows us.

As the other half reaction of overall solar water splitting, the four-electron oxygen evolution reaction (OER) with higher reaction barriers is more difficult to realize than HER. The photocatalytic water oxidation performance of CFHoMSs was evaluated using 0.01 M AgNO_3_ as the sacrificial agent under the same 300 W Xe lamp irradiation. As shown in Fig. 5a, among the CeO_2_-CeFeO_3_ related samples, the maximum O_2_ evolution rate of 452 μmol/h was achieved by 3S-CFHoMSs, which also processes the highest light absorption ability (Supplementary Fig. 8b). We further checked the photocatalytic durability of the corresponding samples by the repeated purge and injection cycles with an interval of two hours. As shown in Supplementary Fig. 8d, the O_2_ evolution rates of all these samples show gradual decrease, and the overall durability of the most active sample 3S-CFHoMSs is less than five hours. Nevertheless, both the oxygen evolution rate and stability of our photocatalyst are outstanding among transition metal oxide- and ceria-based photocatalyst materials (Supplementary Table 5).

The evolution of H_2_ and O_2_ from 100 mL water with the aid of redox shuttle under Xe lamp irradiation is shown in Fig. 5b, in which the calculated H_2_ and O_2_ production in 10 hours is ∼98 and ∼46 μmol, respectively. Activity under simulated sunlight irradiation was also confirmed in Supplementary Fig. 12. Impressively, no production was detected using TiO_2_-Cu_x_O and defect rare CeO_2_-CeFeO_3_ nanoparticles, but H_2_ and O_2_ were continuously evolved in the HoMSs system under the same reaction condition, which is attributed to the structure design. The hollow multi-shell structures greatly increased the light-harvesting ability of photocatalysts, and we believe the charge transportation and surface properties improved by introducing HoMSs into the photocatalytic system also significantly drove the reaction. The comparison of both XPS spectra of Cu 2p for TCHoMSs and Ce 3d for CFHoMSs before and after the overall water splitting reaction have confirmed that the chemical composition remained stable during the photocatalytic reaction (Supplementary Fig. 13).

## DISCUSSION

As confirmed by the characterizations, the design and synthesis of heterostructural HoMSs with the ability of sequential harvesting of light were realized for the first time. The aforementioned experimental results clearly indicate that 4S-TCHoMSs and 3S-CFHoMSs exhibit impressive enhanced light-harvesting ability. Choosing water splitting as a model reaction, the perfectly designed HoMSs can significantly improve the light utilization and bring a great enhancement in catalytic performance.

First, our new concept of sequential absorption of light from outer shell to the inner shells and from the outer part of individual nanoparticle subunit to its inner part, can efficiently improve light absorption, reduce light-to-heat conversion and decrease charge carrier recombination, thus significantly improving light conversion efficiency. The outside-in gradually increased Cu/Ti ratio on the shells enables the sequential absorption of weakly penetrable short-wavelength light by the outer shell and strongly penetrable long-wavelength light by the inner shells, thus widening the light absorption spectrum and strengthening the light absorption ability. The oxygen vacancies and CeFeO_3_ mainly exist on the surface of CFHoMSs, which enable each nanoparticle subunit to sequentially harvest light from the edge to the inside, thus rendering CFHoMSs with visible light response and broadening the action spectrum.

Second, the enhanced photocatalytic water splitting performance was achieved by 4S-TCHoMSs and 3S-CFHoMSs with enhanced sequential light-harvesting ability and additional structural advantages: (1) thin shells shorten the diffusion paths of photogenerated carriers and facilitate electron-hole separation (Supplementary Fig. 14); (2) the aerophobic surface of the hollow structures favors the water absorption and gas desorption, thus improving the kinetic rate of the surface reaction (Supplementary Fig. 15); and (3) a heterostructure of TiO_2_ and Cu_x_O with the unique feature is created (0D nanoparticles make up 3D shells and support each other to construct 3D hollow structures) and greatly lowers both the interfacial and bulk charge-transfer resistance (Supplementary Fig. 16).

## CONCLUSION

In summary, inspired by nature, by simulating the structure and function of cyanobacteria, the sequential harvesting of solar light was successfully realized by well-designed heterogeneous HoMSs with distinct shells composed of different valance-state particles, paving a new route to enhancing light-harvesting efficiency. More impressively, the multiple merits of sequential light harvesting, including enhanced light absorption and inhibited charge carrier recombination, were experimentally verified by a model reaction of water splitting photocatalyzed by purpose-designed heterogeneous HoMSs: 4S-TCHoMSs achieved excellent HER activity of 2490 μmol/h, while 3S-CFHoMSs exhibited impressive OER activity of 452 μmol/h, which are among the best reported so far. This new wisdom of sequential harvesting of solar light or other electromagnetic waves with heterogeneous HoMSs may pave a new way for promoting the development in all photo-related fields as well as microwave absorption areas.

## Supplementary Material

nwaa059_Supplemental_File
