# Supplementary material for: Efficient sequential harvesting of solar light by heterogeneous hollow shells with hierarchical pores
Source: Natl Sci Rev. 2020 Apr 8;7(11):1638–46. doi: 10.1093/nsr/nwaa059 (PMC8290956; doi:10.1093/nsr/nwaa059)
Supplement: nwaa059_Supplemental_File [file nwaa059_supplemental_file.docx]

**Efficient Sequential Harvesting of Solar Light by Heterogeneous Hollow Shells**

Yanze Wei^1,2†^, Jiawei Wan^1†^, Nailiang Yang^1,6†^, Yu Yang^1^, Yanwen Ma^3^, Songcan Wang^4^, Jiangyan Wang^1,^^§^, Ranbo Yu^2^, Lin Gu^5^, Lianhui Wang^3^, Lianzhou Wang^4^, Wei Huang^3*^ and Dan Wang^1,6*^

^1^ State Key Laboratory of Biochemical Engineering, Institute of Process Engineering, Chinese Academy of Sciences, 1 North 2nd Street, Zhongguancun, Haidian District, Beijing 100190, China.

^2^ Department of Physical Chemistry, School of Metallurgical and Ecological Engineering, University of Science and Technology Beijing, 30, Xueyuan Road, Haidian District, Beijing 100083, China.

^3^ School of Materials Science & Engineering, Nanjing University of Posts and Telecommunications, No.9 Wenyuan Road, Nanjing 210046, P. R. China.

^4^ School of Chemical Engineering and Australian Institute for Bioengineering and Nanotechnology, The University of Queensland, St Lucia
Queensland 4072 Australia

^5^ Institute of Physics, Chinese Academy of Sciences, No. 8, 3rd South

Street, Zhongguancun, Beijing 100190, China

^6^ University of Chinese Academy of Sciences, Chinese Academy of Sciences,

No. 19A Yuquanlu, Beijing 100049 (China)

† These authors contributed equally to this paper.

§ Current address: Department of Materials Science and Engineering, Stanford University, Stanford, California 94305, USA.

**Methods**

**Synthesis of TiO_2_-CuO_x_ hollow multi-shelled structures (TCHoMSs).** All chemicals were bought from Beijing Chemical Reagent Factory and used without further treatment. Sequential Templating Method was employed to obtain controlled multi-shelled hollow spheres. Carbon microspheres (CMSs) were obtained using the method we reported before and they were employed as the hard template in the further synthesis. Titanium tetrachloride (TiCl_4_) and copper chloride (CuCl_2_∙2H_2_O) were used as precursor offering metal ions. In absorption process, the as-prepared CMSs (0.6 g) were decentralized in the solution of 3 M titanium tetrachloride and 0.24 M copper chloride (60 mL). Then the solution was sonicated in cool water for 30min to remove the agglomeration of CMSs. After that, the solution was stirred in water bath with the temperature of 20-50 ^o^C. Then the mixture was filtered and washed once and dried in 35 ^o^C for 12 h. Multi-shell hollow spheres with different number of shells were obtained with different stirring time and temperature of water bath (20 ^o^C, 3h for 1S-TCHoMS; 20 ^o^C, 12h for 2S-TCHoMS; 50 ^o^C, 12h for 3S-TCHoMS; and 50 ^o^C, 24h for 4S-TCHoMS). After drying, CMSs were placed in porcelaneous boat in the furnace and calcined from room temperature to 400 ^o^C and then held for 120 min with the heating rate of 2 ^o^C/min. At last, the samples were cooled to room temperature naturally.

**Synthesis of CeO_2_-CeFeO_3_ hollow multi-shelled structures (CFHoMSs).** The cage-like CeO_2_-CeFeO_3_ hollow spheres were fabricated via the same sequential templating approach by applying CMSs as the hard template. After soaking CMSs into 10 mL of water, both [cerium nitrate](http://www.baidu.com/link?url=0i3UcEq78ZZUJA5-lTL7nzeySQCK20_i_EL5dfrRzSzLJJa2Mo_5nG7tC1F1G2Y5b4KBwsdnzAooDiuH-9em1bpyoMjgQhLdR3lfIxjB5isQPP6BpzMCTwR4vvB0yQ5z) (0.5 M) and [iron nitrate](http://www.baidu.com/link?url=AN2eQbYn5A335H3VqtKbnvIbQ1FOJ6iRre0_46qUiXuMt6voNKX_DNOjg0peu6fStYH-fvEYnxK3TtDf8_hsBDtjGQJvk0dx-2hA91u_Owa9iZXmQPGROXH8yIkS9tTs) (0.005 M) were dissolved as the precursors. Then 20 mL of ethanol was added into the aqueous solution and the mixture was stirred at certain temperature for different times (20 ^o^C, 12h for single shell hollow structures; 40 ^o^C 12h for double shell hollow structures; and 40 ^o^C 24h for triple shell hollow structures). Multi-shell hollow structures were obtained by sintering in the muffle furnace from room temperature to 450 ^o^C and held for 2 h. In the post treatment procedure, the samples were calcined at O_2_ flow at 800 ^o^C for 1h and then changed to Ar-H_2_ (10% H_2_ in volume) flow and kept at 800 ^o^C for another 5h to get CFHoMSs. The defect-rare CFHoMSs were further treated using O_2_ under 800 ^o^C for 2h.

**Synthesis of** **TiO_2_-Cu_x_O NPs.** The synthesis of TiO_2_-Cu_x_O NPs (TCNPs) was achieved by a simple sol-gel method. Certain amount of titanium tetrachloride was dispersed in Cu(NO_3_)_2_ solution with PEG-6000, and then the mixture was stirred for 1h. After that, sucrose and NH_3_·H_2_O solution were added into the solution in 90 ^o^C and stirred for another 1h to evaporate the excess water. Finally, the as-prepared solid was dried under 100 ^o^C for 6h and calcined at 400 ^o^C for 2h in muffle oven. The sample was stored in centrifuge tube in the desiccator to avoid water vapor.

**Materials characterization.** TEM, HRTEM and SAED images were obtained on a JEOL JEM-2100F instrument (JEOL, Japan) with the accelerating voltage of 200 kV. Spherical aberration-corrected STEM was tested through a JEOL 2100F (JEOL, Japan) transmission electron microscope with a CEOS (Heidelberg, Germany) probe aberration corrector, operated at 200 kV. SEM images were obtained through JSM-6700 microscope (5.0 kV). Powder XRD patterns were achieved through a Panalytical X’ Pert PRO MPD [Cu Ka radiation (λ = 1.5405 Å), 40 kV, 30 mA] with a scan rate of 10^o^ per minute. XPS data were collected by an ESCALab220i-XL electron spectrometer (VG Scientific, USA) with 300 W Al Ka radiation. The binding energies were internally standardized with respect to the C 1s (284.8 eV). UV-Vis diffuse reflection absorption spectra (UV-Vis/DRS) of the samples were recorded by a Cary 5000 UV-Vis spectrometer (Varian, USA) equipped with an integrating sphere accessory and BaSO_4_ as reference material. The sample weight was 25 mg, which was uniformly distributed in an area of about 1.5 cm^2^ in size. PL intensity of different samples were studied by a FLS980 lifetime and steady state spectrometer (EI, UK) with the excitation wavelength 340 nm for TCHoMS and 366 nm for CFHoMS. Contact angles (CA) were measured on an OCA20 system (Data-Physics, Germany). For liquid-solid contact angles, water droplets (2 μL) were syringed out and dropped carefully onto the surfaces, which were measured at five different positions on the same sample. For gas-solid contact angles, gas bubbles (air, 2 μL) were syringed out and attached on to the surface, which were immersed in the water. The adhesion forces were measured on a high-sensitivity micro-electromechanical balance system (DCAT 11 system, Data-Physics, Germany) and measured in a water environment. Typically, a gas bubble (about 6 μL) floating on a metal ring was approached and retracted from the sample surface at a constant speed of 0.05 mm·s^−1^ at ambient environment. The bubble started to move away from the sample surface once contacting, and the balance force would gradually increase, and reached the maximum before the bubble leave from the surface. The peak data recorded in the force-distance curve was taken as the maximum adhesion force.

**Water splitting performance measurement.** Solar water splitting for hydrogen or oxygen evolution performance were carried out in the water splitting online analysis system (LabSolar 6A, Beijing Perfectlight Technology Co., Ltd.,) with the 300W Xe lamplight source (light intensity: 600 mW/cm^-2^). For half reaction, 25 vol% methanol was used as the sacrificial reagent for HER activity evaluation while 0.01 M AgNO_3_ was used as the OER sacrificial reagent. The samples for HER and OER were weighted to be 180 mg and 160 mg, respectively. The condensed water circulation system was operated at 281 K using water as circulating fluid. The evolved gas was analyzed by gas chromatograph (GC-7900, Techcomp, China, TCD, Ar as the carrier gas and 5A molecular sieve column). For durability test, every cycle was carried out for 2 h followed by centrifugation and washing with DI water. The average intensity of irradiation was by an S310C spectroradiometer (THORLABS) and the details were shown in Supplymentary Table 3. The apparent quantum efficiency (AQE) was measured under the identical photocatalytic reaction. The AQE was calculated according to the following equation (1):

AQE$=\frac{Number of reacted electron}{Number of incident photons}\times100$%

$=\frac{Number of evolved H2 molecules \times2}{Number of incident photons}\times100\%$ (1)

For overall water splitting, the HER photocatalyst was photo-deposited with 2 wt% Pt while the OER photocatalyst was photo-deposited with 1 wt% Ru, as described elsewhere. The FeCl_3_ solution with pH adjusted to be 2 by sulfuric acid was used as the reactant solution to provide Fe^3+^/Fe^2+^ electron mediator in the Z-scheme system. Measurements were carried out in the water splitting online analysis system (LabSolar 6A, Beijing Perfectlight Technology Co., Ltd.,) with the simulated sunlight as the light source. The temperature of the system was kept at 281 K using a water bath. The amounts of H_2_ and O_2_ produced were determined using gas chromatograph (GC-7900, Techcomp, China, TCD, Ar as the carrier gas and 5A molecular sieve column).


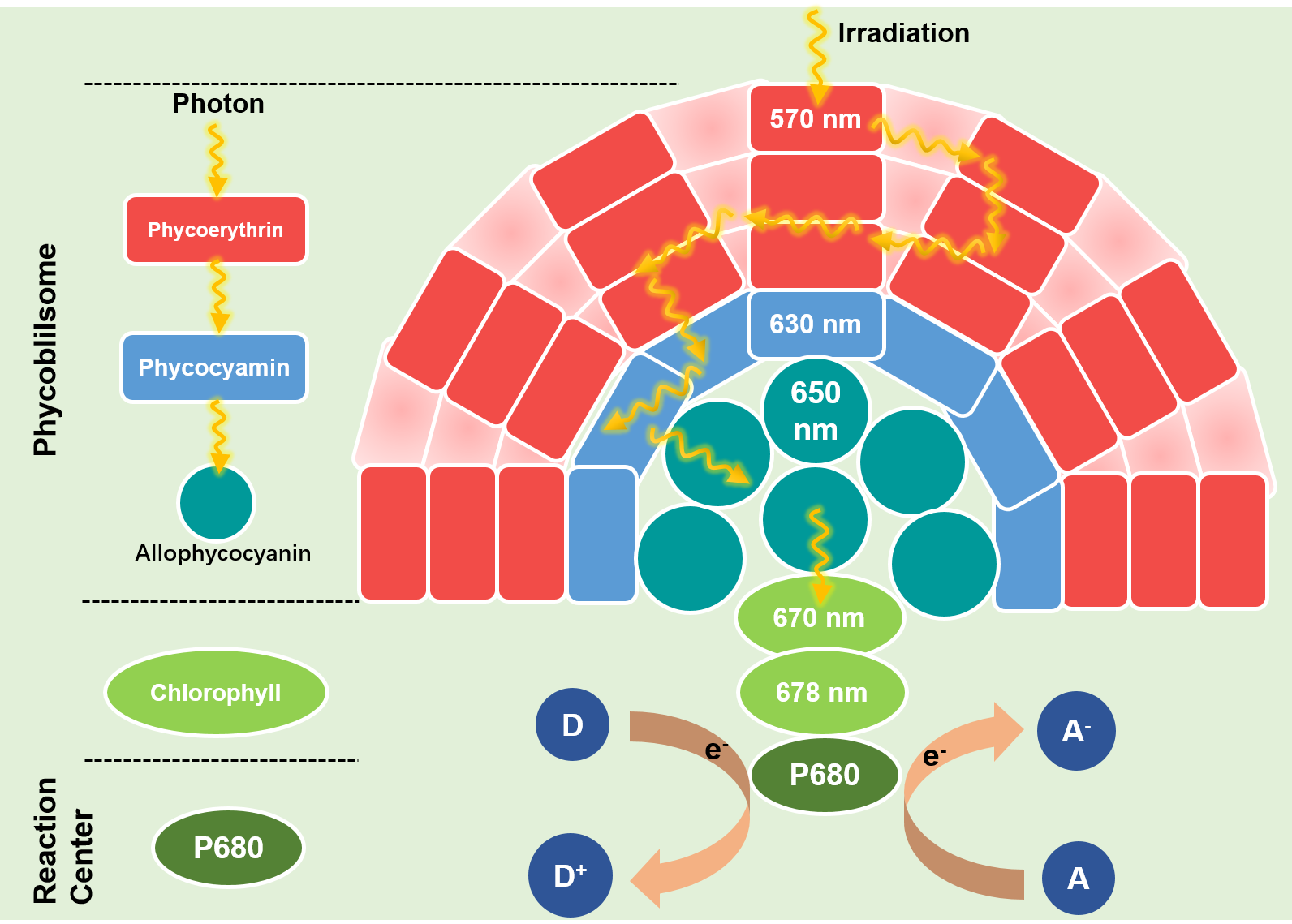


**Supplementary Figure 1︱**Structural organization of the antenna system for red algae and cyanobacteria. The incident light is efficiently harvested by passing through a series of antenna pigments. The wavelength numbers (nm) inside the circles represent the absorption edge of these pigments. The above mentioned structure of antenna system is accordance with the review article “Adventures with cyanobacteria: a personal perspective” (Govindjee, *Frontiers in Plant Science*, 2011, 2, 28.).


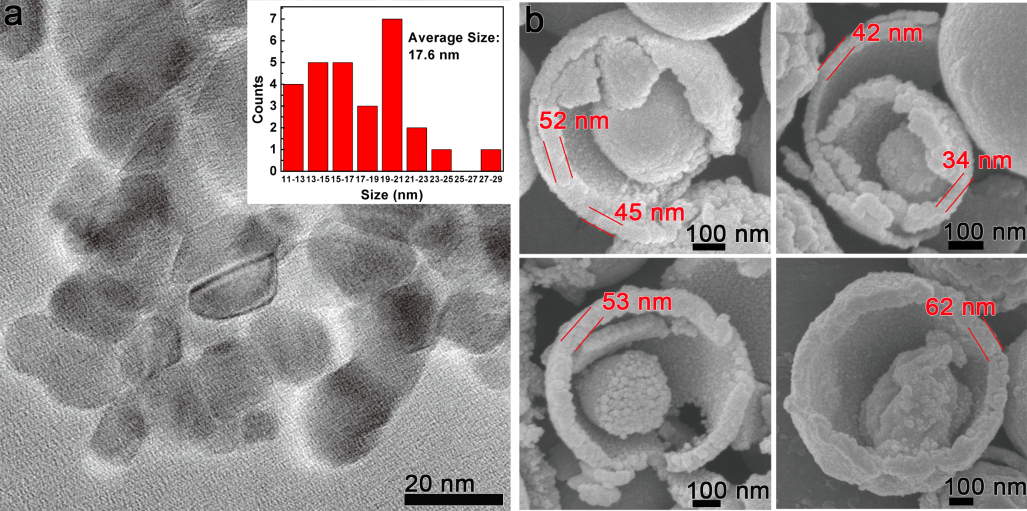


**Supplementary Figure 2︱**(a) TEM image of composited shell in TCHoMSs. (b) Thickness of different shells of TCHoMSs.


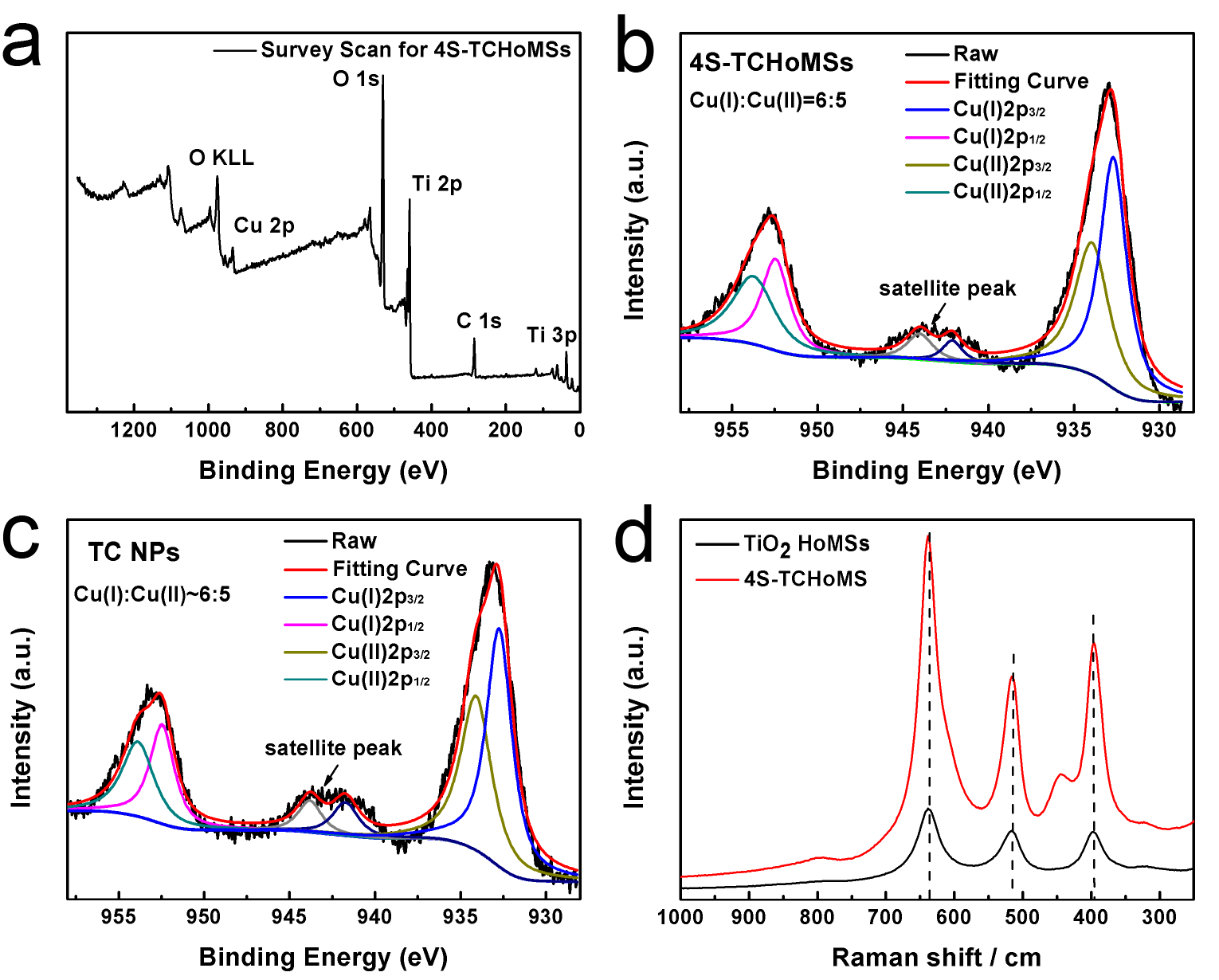


**Supplementary Figure 3︱Chemical composition of TCHoMSs and TCNPs:** (a) XPS survey spectrum of 4S-TCHoMSs; (b) The high-resolution XPS spectrum of Cu 2p of 4S-TCHoMSs; (c) The high resolution XPS spectrum of Cu 2p of TiO_2_-Cu_x_O nanoparticles; and (d) Laser Raman spectra of 4S-TCHoMSs and pure TiO_2_ HoMSs.


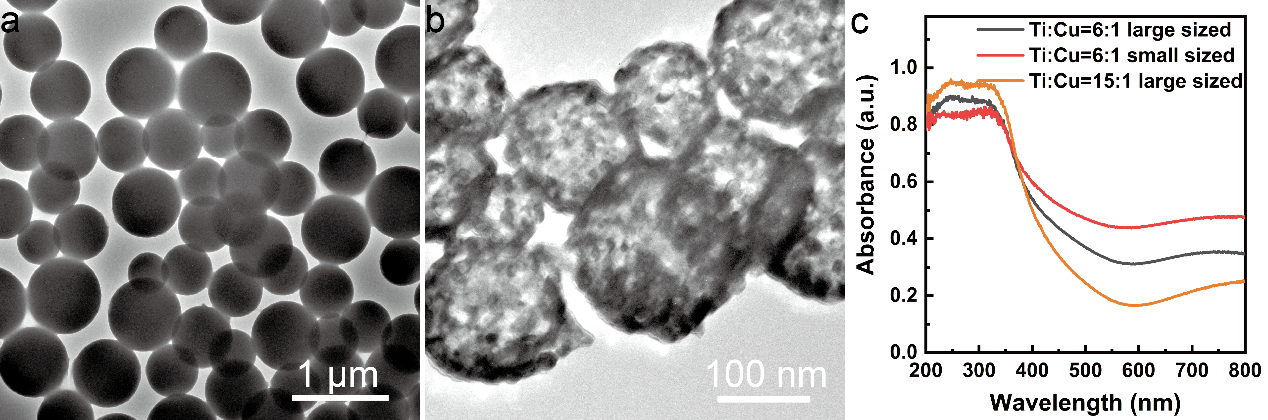


**Supplementary Figure 4︱Light absorption of TiO_2_-Cu_x_O single-shelled hollow spheres (Ti/Cu=6:1) with different sizes**. (a) Carbon templates used for fabricating small sized TiO_2_-Cu_x_O single-shelled hollow spheres. (b) TEM image of obtained TiO_2_-Cu_x_O single-shelled hollow spheres (with measured molar ratio of Cu/Ti to be 0.153 by ICP-MS). (c) UV-Vis spectra of TiO_2_-Cu_x_O single-shelled hollow spheres with different sizes and molar ratios.

In detail, to inspect the size-effect on hollow spheres with Ti/Cu molar ratio of 6:1, extra experiments have been conducted and the results are shown in **Supplementary Fig. 4**. To mimic the hollow spheres as the inner shell of 4S-TCHoMSs, smaller carbon templates are applied, as shown in **Supplementary Fig. 4a**. Following similar STA strategy, the smaller sized TiO_2_-Cu_x_O single-shelled hollow spheres (d=120-150 nm, STCHSs) are obtained (**Supplementary Fig. 4b**). The Ti/Cu ratio is adjusted to be 6:1, which is the same as the larger sized TiO_2_-Cu_x_O single-shelled hollow spheres (～600 nm, LTCHSs) reported in the paper. Indeed, the size of hollow structure affects the light absorption ability. The smaller hollow sphere prefers to absorb the visible light, while the larger hollow sphere has its advantage in the UV light absorption (**Supplementary Fig. 4c**). This result is consistent with our designation for sequential light absorption of TCHoMSs, i.e., the inner shell has the advantage for the absorption of light with the longer wavelength, while the outer for the shorter ones.

Notably, we should mention, although the size has affected the light absorption, the dominated light response is contributed by the composition. When comparing with the large hollow sphere of TiO_2_-Cu_x_O with different Ti/Cu molar ratio (6:1 and 15:1), it shows the sphere with a larger Cu content, the stronger light absorption at the longer wavelength is achieved.


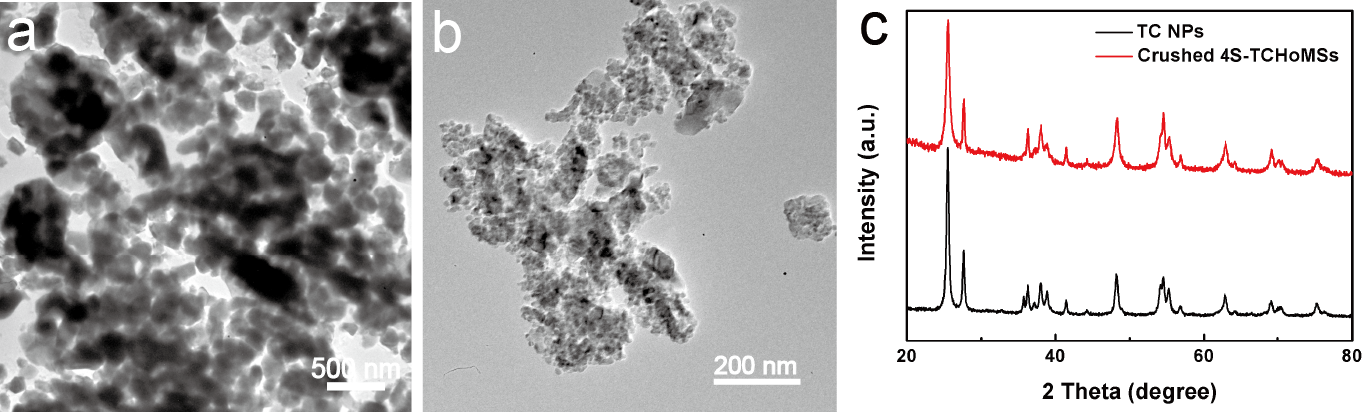


**Supplementary Figure 5︱**TEM images of (a) TCNPs and (b) crushed 4S-TCHoMSs. (c) XRD patterns of TCNPs and crushed 4S-TCHoMSs.


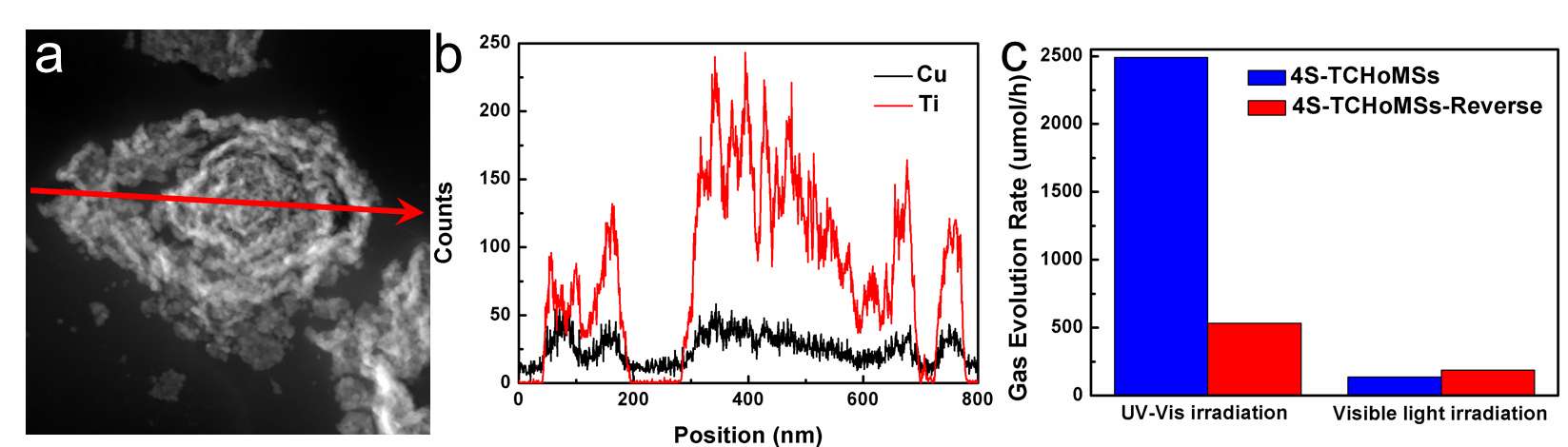


**Supplementary Figure 6︱**(a) TEM image of a slice of reversed 4S-TCHoMSs in dark field, b) Ti/Cu ratios in different shells by EDS line scanning, and (c) photocatalytic HER performance of 4S-TCHoMSs and reversed 4S-TCHoMSs.

TCHoMSs with reversed TiO_2_ and Cu_x_O compositions were synthesized through a special adsorption procedure. The carbonaceous templates were first soaked in the Ti rich solutions and then soaked in Cu rich solutions. The ratios of Ti and Cu in the slice of reversed TCHoMSs were confirmed by X-Ray spectroscopy (EDS) line-scanning characterization. Results in **Supplementary Fig. 6a and b** confirmed that more Ti element existed in the inner shells. The related photocatalytic performance proved the sequence is very important in HoMSs. As shown in **Supplementary Fig. 6c**, although the reversed TCHoMSs showed slightly enhanced visible light driven photocatalytic HER performance (the content of Cu is increased in the outer shells), the performance under UV-Visible light irradiation decreased to only one fifth of the 4S-TCHoMSs as demonstrated in the manuscript. The result verified that our novel design of the outmost shell composed of broad-bandgap materials to absorb weak-penetrable short wavelength light, while the inner shells composed of increased ratio of narrow-bandgap materials to absorb strong-penetrable long wavelength light, enables sequential light harvesting and significantly enhances the total light harvesting and improves the photocatalytic performance.


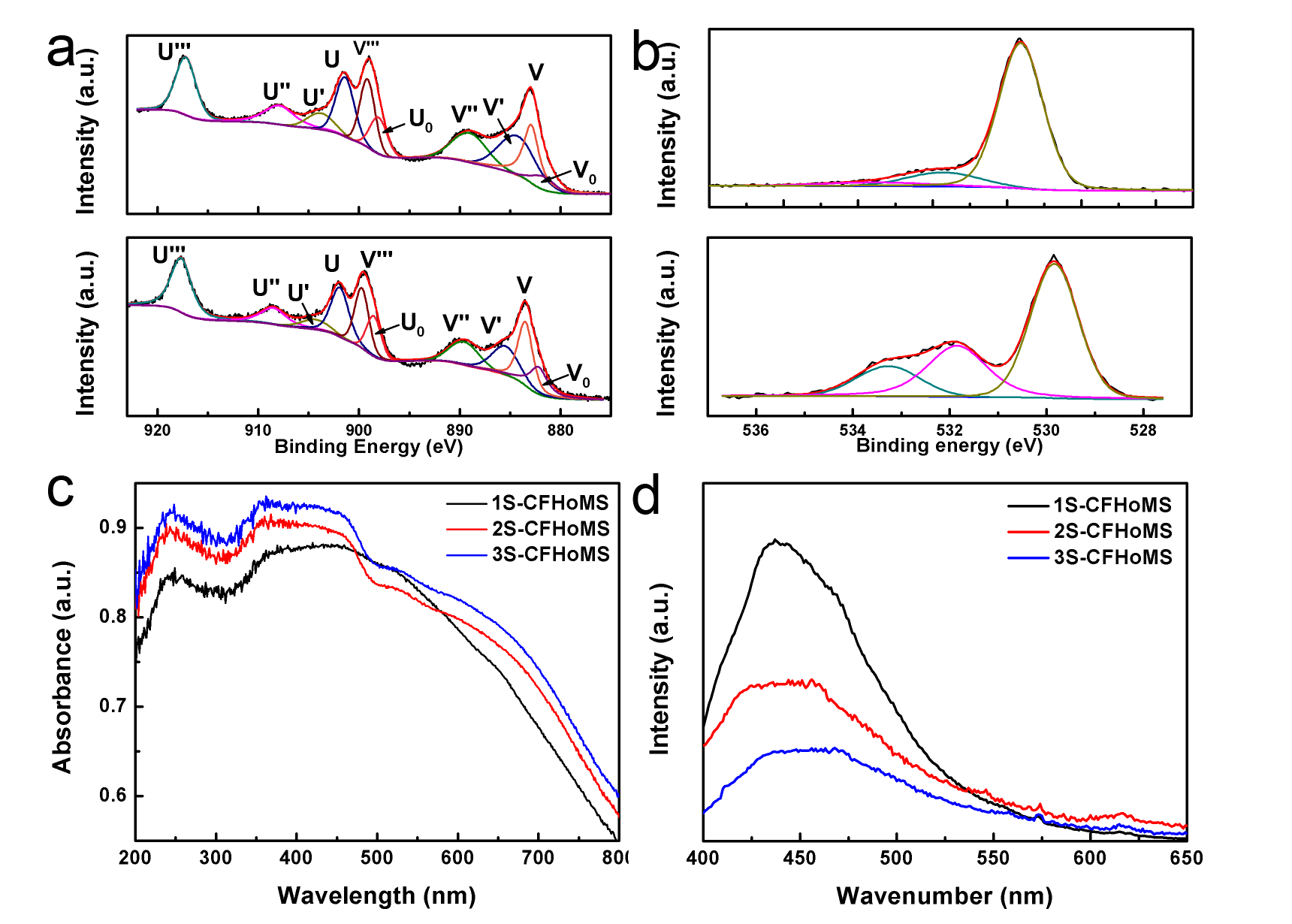


**Supplementary Figure 7︱**(a,b) high resolution XPS spectra for Ce 3d and O 1s orbits with upper row for CeO_2_-Fe_2_O_3_ hollow spheres and lower row for reduced 3S-CFHoMS (U''', V''', U'', V'', U, V, U', V', U_0_, V_0_ represent the peaks of Ce^4+^ 3d_5/2_, Ce^4+^ 3d_3/2_, Ce^4+^ satellite, Ce^4+^ satellite, Ce^4+^ satellite, Ce^4+^ satellite, Ce^3+^ 3d_5/2_, Ce^3+^ 3d_3/2_, Ce^3+^ satellite and Ce^3+^ satellite, respectively). (c) UV-Vis absorption curves of different shelled CeO_2_-CeFeO_3_ hollow spheres; (d) PL spectra for different shelled CeO_2_-CeFeO_3_ hollow spheres.


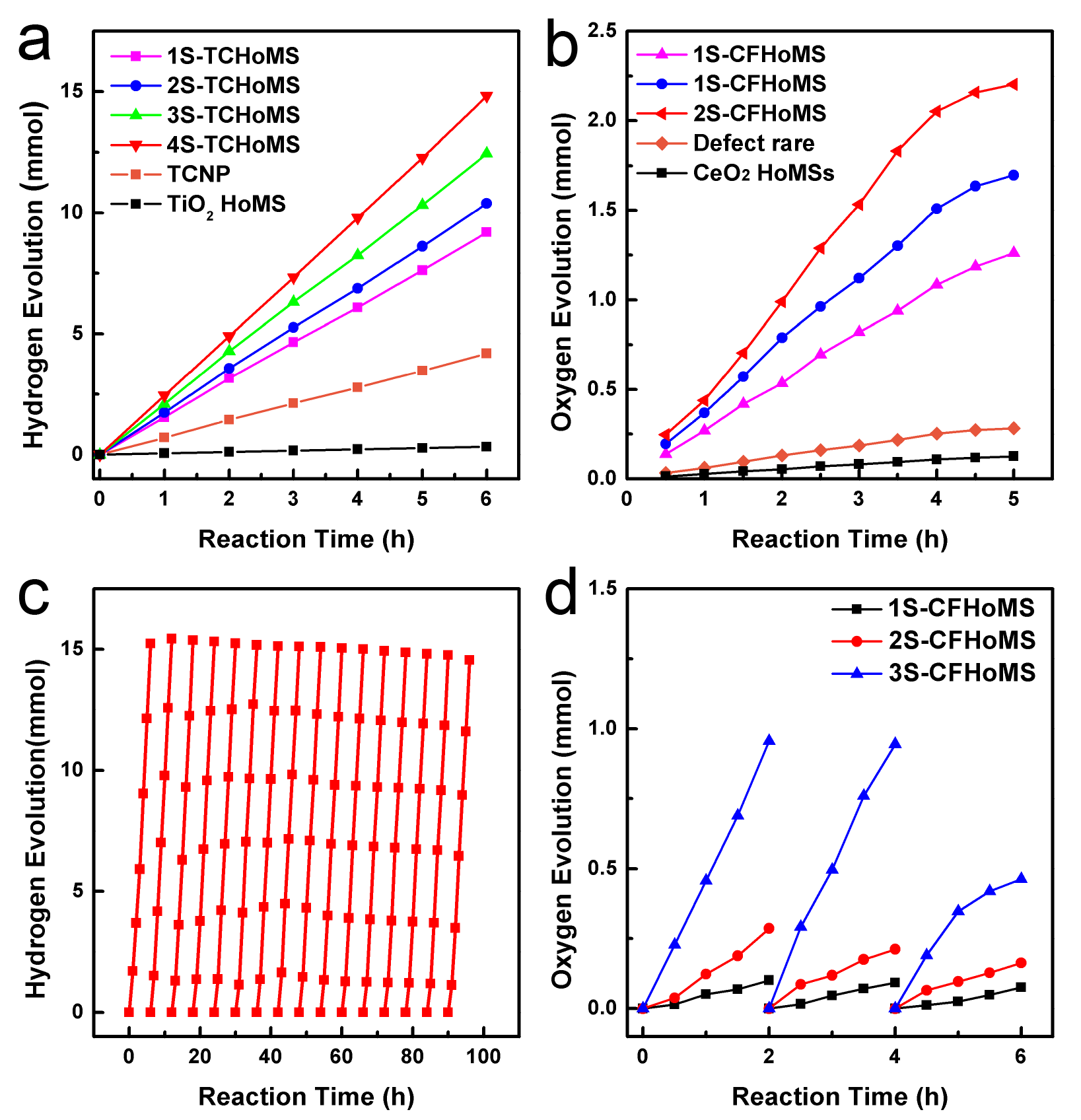


**Supplementary Figure 8︱** (a) Hydrogen evolution activity of TCHoMSs with different number of shells under 300 W Xe lamp at 281 K with 180 mg of photocatalysts, (b) Oxygen evolution activity and stability of CFHoMSs under 300 W Xe lamp at 281 K with 160 mg of photocatalysts, (c) Hydrogen evolution stability of TCHoMSs with different number of shells under 300 W Xe lamp at 313 K and 1 kPa with 180 mg of photocatalysts; and (d) Oxygen evolution stability of CFHoMSs under 300 W Xe lamp at 313 K and 1 kPa with 160 mg of photocatalysts.


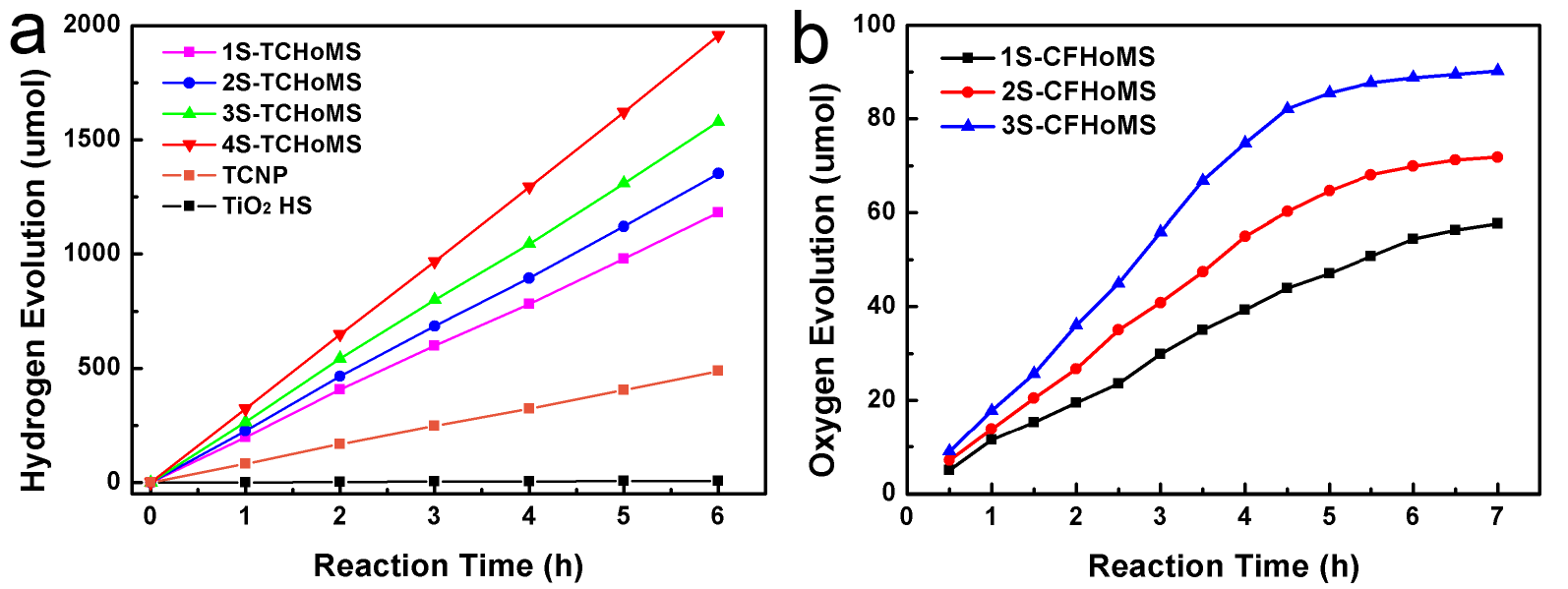


**Supplementary Figure 9︱**(a) HER and (b) OER activity of TCHoMS and CFHoMS with different number of shells under simulated sunlight (AM 1.5G) with light intensity of 100 mW/cm^2^ at 288 K and 1 kPa.


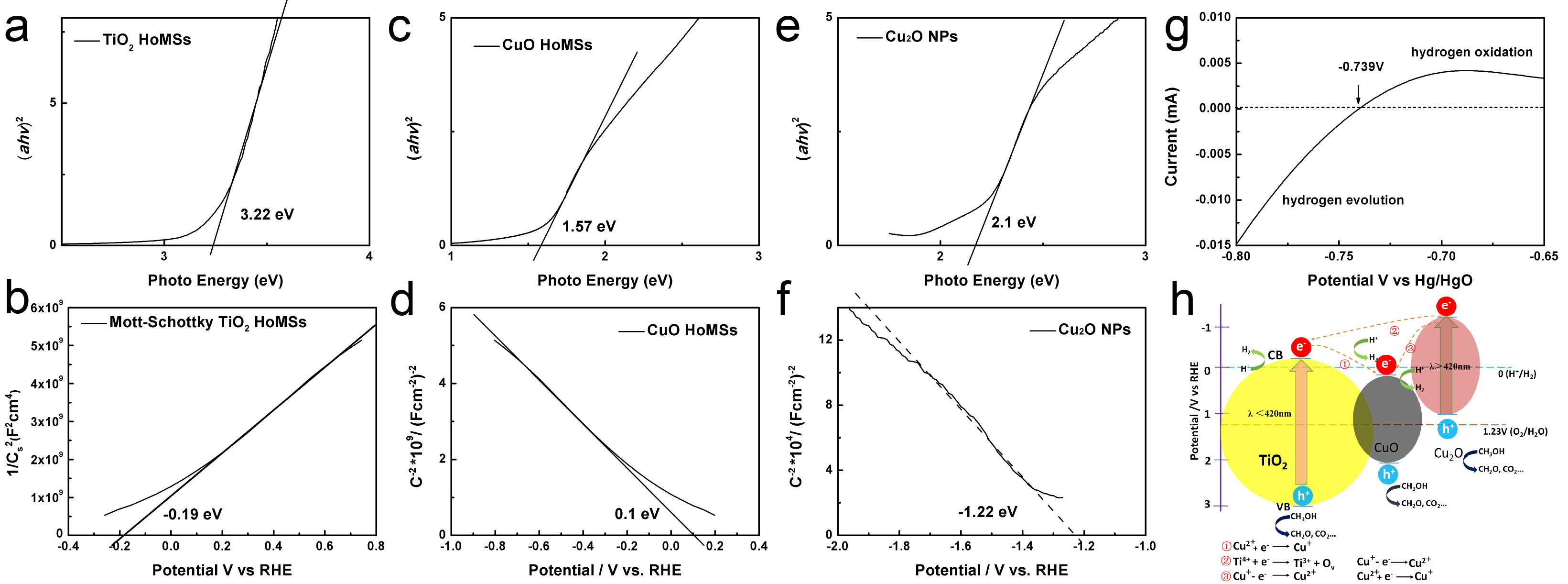


**Supplementary Figure 10︱**Tauc plots and Mott-Schottky plots of (a,b) TiO_2_ HoMSs; (c,d) CuO HoMSs; and (e,f) Cu_2_O NPs, respectively; (g) Calibration of Hg/HgO reference electrode, and (h) illustration of possible paths of electron transportation in TCHoMSs.


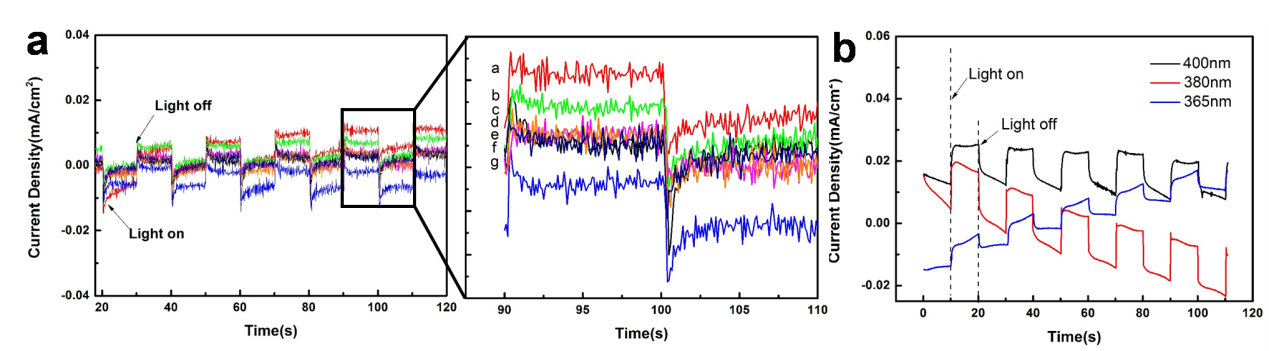


**Supplementary Figure 11︱**Chopped light *J-t* curves of 4S-TCHoMS photocatalyst under light with different wavelength: (a) 420 nm, 450 nm, 475 nm, 500 nm, 520 nm, 550 nm, 600 nm (insert photo from a to g); and (b) 365 nm, 380 nm and 400 nm.

To elucidate the electron transport path of the 4S-TCHoMSs photocatalyst, the output photocurrent responses were investigated by varying the incident light wavelength and utilizing an optical chopper. Under visible light illumination (**Supplementary Fig. 11a**), a negative output photocurrent was observed, which corresponding to a p-type semiconductor photo response behavior. On the other hand, upon UV illumination (**Supplementary Fig. 11b**), positive output photocurrent revealed a photoresponse induced by the n-type semiconductor. Results obtained from the J-t curve under chopped light suggest that the Cu_2_O phase in the catalyst would generate electrons and holes upon visible light illumination with λ>420 nm and the existing TiO_2_ or CuO composite could construct a type II heterojunction with Cu_2_O. Besides, under UV illumination (λ<420 nm), TiO_2_ was excited by photons and the type II heterojunction formed by TiO_2_ and CuO facilitated the electron-hole separation process, thus enhancing the photocatalytic performance.





**Supplementary Figure 12︱**Overall water splitting performance of TCHoMS and CFHoMS. Catalyst: 100 mg of each catalyst, reaction solution: 150 mL of 2 mM FeCl_3_ aqueous solution, reaction cell: a top-window reaction cell, light source: AM 1.5G (100mW/cm^2^).

The evolution of H_2_ and O_2_ from 100 mL water by applying 2 mM FeCl_3_ as the redox shuttle under simulated sunlight irradiation is shown in Supplementary Fig. 8. Impressively, H_2_ and O_2_ were both quantified by gas chromatography (GC) and evaluated continuously in a molar ratio of 2.03, effectively equal to the theoretical value of 2 for overall water splitting. The calculated H_2_ evolution production was up to ~15 μmol and that of O_2_ ~7.5 μmol in 10h, and no other gases except H_2_ and O_2_ were detected by GC. Notable, as for the TiO_2_-Cu_x_O and CeO_2_-CeFeO_3_ based nanoparticles, no production was detected in the same reaction condition, and we believe the enhanced light harvesting of HoMSs greatly drove the reaction. The comparison of both XPS spectra of Cu 2p for TCHoMSs and Ce 3d for CFHoMSs before and after the overall water splitting reaction have confirmed that the chemical composition remained stable during the photocatalytic reaction.

The water splitting reaction was carried out under simulated sunlight illumination. The STH is given as:

STH (η) =$\frac{Output energy as H_{2}}{Energy of incident solar lihgt} \times100\%$= $\frac{\Delta G\times r_{H_{2}}}{P_{sun}\times S'}\times100\%$

= $\frac{237\frac{kJ}{mol}\times1.5\times\frac{{10}^{-3}mol}{h}}{1000W/m^{2}\times1.65\times{10}^{-3}m^{2}}\times100\%$= 0.006%

Where ΔG, r_H2_, P_sun_ and S’ denote the reaction Gibbs energy of the water splitting reaction (237 kJ/mol), the rate of hydrogen evolution during the water splitting reaction, the energy intensity of the AM 1.5G solar irradiation (100 mWcm^-2^) and the irradiated sample area (16.5 cm^2^), respectively.

Comparisons of performance with other photocatalysts were listed in **Supplementary Table 6**.


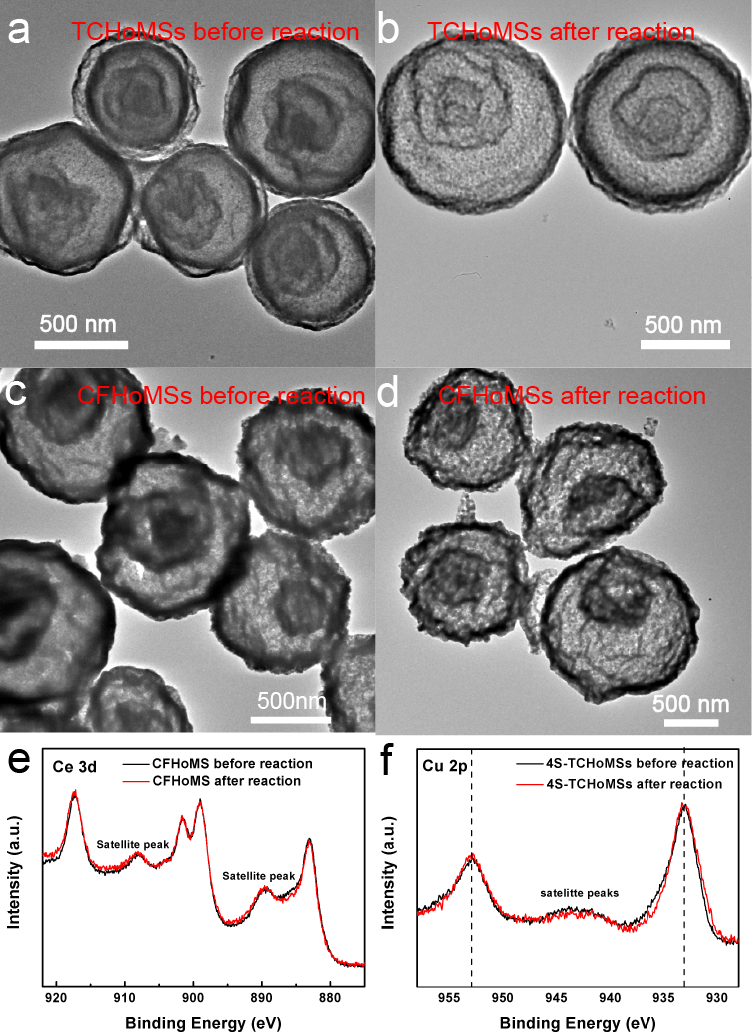


**Supplementary Figure 13︱**TEM images of (a,b) TCHoMSs and (c,d) CFHoMSs before and after overall water splitting reaction; XPS spectra of (e) Ce 3d for CFHoMS and (f) Cu 2p for TCHoMS before and after the overall water splitting reaction.





**Supplementary Figure 14︱**PL spectra of commercial TiO_2_-Cu_x_O nanoparticles and TCHoMSs with different shell numbers.

The photoluminescence (PL) intensity of TCHoMSs samples is lower than that of TCNPs, indicating that the TiO_2_-Cu_x_O heterostructures can effectively suppress the charge recombination. Moreover, the PL intensity decreases as shell number increases, which further demonstrates that the thin shells shorten the diffusion paths of photogenerated electrons and holes. Thus charge carriers can reach shell surface for reactions more easily, resulting in enhanced charge separation and inhibited charge recombination.


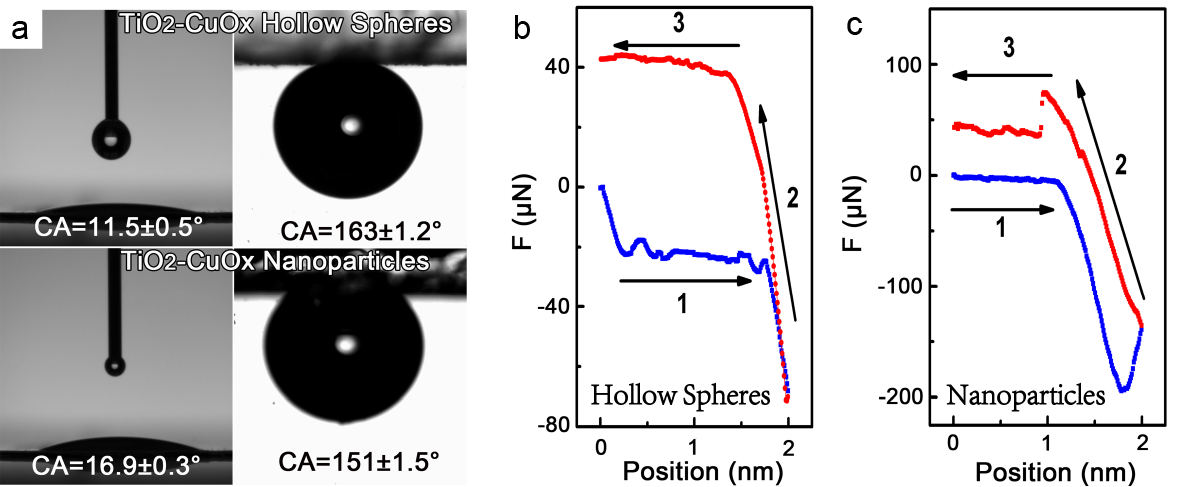


**Supplementary Figure 15︱(**a) contact angle test of 4S-TCHoMSs (upper row) and TCNPs (lower row); and adhesive forces measurements of (b) 4S-TCHoMSs and c) TCNPs.


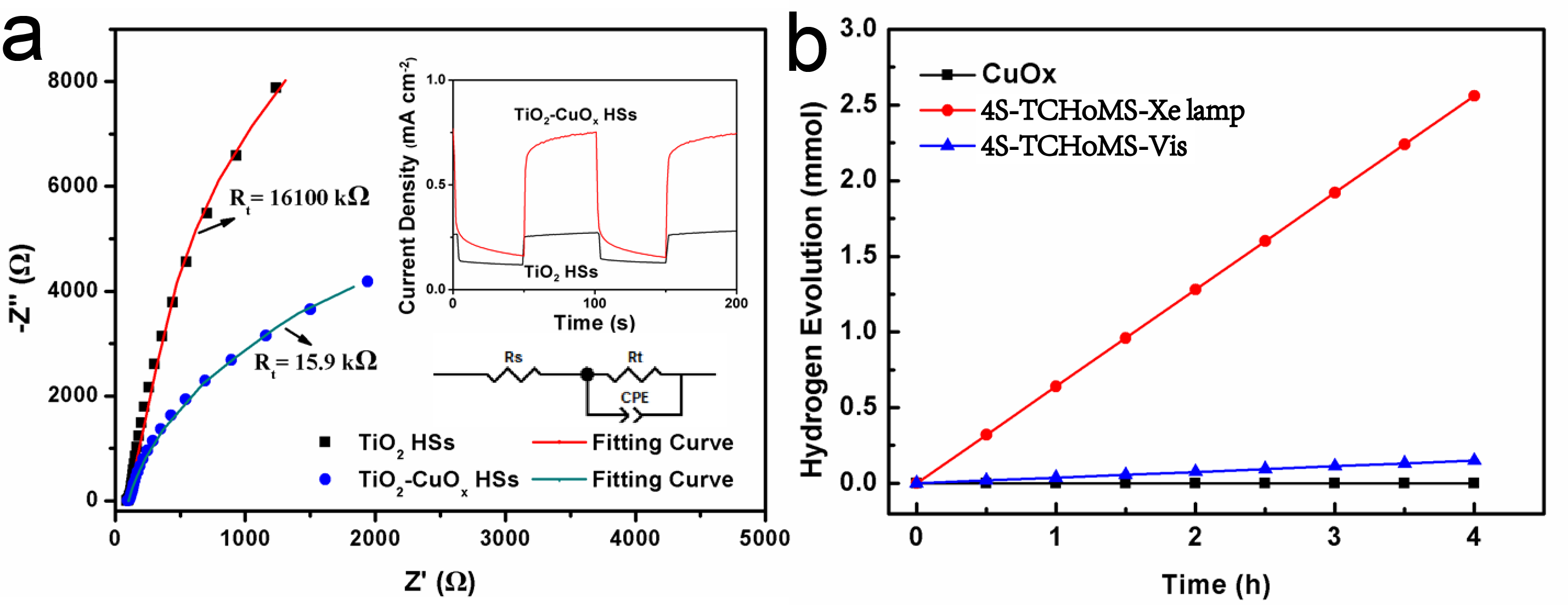


**Supplementary Figure 16.** (a) EIS Nyquist plots of TiO_2_ hollow spheres and TiO_2_-Cu_x_O hollow spheres measured under the open-circle potential and light irradiation in 0.5 M potassium phosphate buffer (pH=7) solution. The insert shows the transient photocurrent responses of two electrodes in 0.2 M Na_2_S+0.04 M Na_2_SO_3_ mixed aqueous solution under light irradiation. (b) HER reactivity of Cu_x_O hollow spheres and TCHoMS (0.01 g sample).

EIS measurements were performed on an electrochemical analyser (CHI760D instruments) in a standard three-electrode system utilizing the synthesized samples as the working electrodes, Ag/AgCl (saturated KCl) as a reference electrode, and a Pt wire as the counter electrode. The EIS were recorded over a range from 1 to 10^5^ Hz with AC amplitude of 0.02 V. Photocurrent was measured in the same three-electrode system. A 300 W Xenon light was applied as the light source.

**Supplementary Table 1.** Inductively coupled plasma mass spectrometry (ICP-MS) results of multi-shelled TiO_2_-Cu_x_O hollow spheres and CeO_2_-CeFeO_3_ multi-cages.

|  | Single | Double | Triple | Quadruple | Nanoparticles |
| --- | --- | --- | --- | --- | --- |
| Molar ratio of Cu/Ti | 0.065 | 0.066 | 0.068 | 0.071 | 0.067 |
| Molar ratio of Fe/Ce | 0.058 | 0.059 | 0.061 | N/A | N/A |

**Supplementary Table 2.** Inductively coupled plasma mass spectrometry (ICP-MS) results of single shell TiO_2_-Cu_x_O hollow spheres with specific molar ratio.

|  | 1/15 outer most single shell structures | 1/6 inner most single shell structures |
| --- | --- | --- |
| Measured molar ratio of Cu/Ti | 0.063 | 0.158 |

**Supplementary Table 3.** Calculated apparent quantum Efficiency (AQE) at different wavelengths with irradiated sample area of 16.5 cm^2^.

| Sample | Wavelength | H_2_/O_2_ Evolved | Light Intensity | AQE |
| --- | --- | --- | --- | --- |
| 4S-TCHoMS | 365 nm | 953 μmol | 16.18 mW/cm^2^ | 16.26 % |
| 4S-TCHoMS | 380 nm | 632 μmol | 14.63 mW/cm^2^ | 11.45 % |
| 4S-TCHoMS | 400 nm | 335.19 μmol | 13.28 mW/cm^2^ | 6.36 % |
| 4S-TCHoMS | 420 nm | 262.19 μmol | 12.67 mW/cm^2^ | 4.96 % |
| 4S-TCHoMS | 500 nm | 126.42μmol | 13.47 mW/cm^2^ | 1.89 % |
| 4S-TCHoMS | 550 nm | 0 | 15.04 mW/cm^2^ | 0 |
| TC NPs | 400 nm | 54.78 μmol | 13.28 mW/cm^2^ | 1.04 % |

Take wavelength λ=365 nm for example:

$N=\frac{E\lambda}{hc}=\frac{16.18\times{10}^{-3}\times4\times3600\times16.5\times365\times{10}^{-9}}{6.626\times{10}^{-34}\times3\times{10}^{8}}$=7$\times{10}^{21}$

AQE=$\frac{the number of reacted electrons}{the number of incident photons}\times100\%$

=$\frac{2\times the number or evolved H_{2} molecules}{N}\times100\%$

=$\frac{2\times6.02\times{10}^{23}\times953\times{10}^{-6}}{7*{10}^{21}}\times100\%=$16.26%

**Supplementary Table 4.** Comparison of hydrogen evolution reaction (HER) performance of TiO_2_ based material.

| Materials | Light source | Light intensity | Reactant solution | Activity  (μmol/h) | Reaction time | AQE  (365nm) | Ref. |
| --- | --- | --- | --- | --- | --- | --- | --- |
| TiO_2_-Cu_2_O | UV-Vis (Hg) | 13000lx | Glycerol | 20060 | 5h | N/A | 1 |
| TiO_2_-Cu_2_O | AM 1.5G | N/A | Methanol | 152.3 | 35h | 7.05% | 2 |
| TiO_2_-MoS_2_ | Vis (λ>420 nm) | N/A | TEOA | 50.2 | 8h | N/A | 3 |
| TiO_2_-MoS_2_ | UV-Vis (Xe) | N/A | Ethanol | 43 | N/A | N/A | 4 |
| TiO_2_-MoS_2_ | Vis (λ>420 nm) | N/A | Methanol | 1.5 | 8h | N/A | 5 |
| TiO_2_-WO_3_-C_3_N_4_ | Vis (λ>420 nm) | N/A | Methanol | 286.6 | N/A | N/A | 6 |
| TiO_2_-CuO/Cu | UV-Vis (Xe) | N/A | Methanol | 85.1 | 8h | N/A | 7 |
| TiO_2_-Cu_2_O | UV-Vis (Xe) | N/A | Methanol | 542 | 25h | 13.5% | 8 |
| TiO_2_-Cu/CuO | AM 1.5G | 100 mW/cm^2^ | Methanol | 7150 | 30h | 10.5% | 9 |
| TiO_2_-CoP | UV-Vis (Xe) | N/A | Methanol | 835 | 5h | 3.8% | 10 |
| Au/TiO_2_–gC_3_N_4_ | Vis (λ>420 nm) | 12.5  mW/cm^2^ | TEOA | 87.5 | 5h | 30%  (visible) | 11 |
| Nb-TiO_2_ | UV-Vis (Xe) | N/A | Methanol | 1000 | 20h | 12.1% | 12 |
| 4S-TCHoMS | UV-Vis (Xe) | 103  mW/cm^2^ | Methanol | 2490 | 90h | 16.26% | this |
| 4S-TCHoMS | Vis (λ>420 nm) | 100  mW/cm^2^ | Methanol | 136 |  | 16.26% | work |

1. Lalitha K, Sadanandam G, Kumari V D, et al. Highly Stabilized and Finely Dispersed Cu_2_O/TiO_2_: A Promising Visible Sensitive Photocatalyst for Continuous Production of Hydrogen from Glycerol: Water Mixtures. *Journal of Physical Chemistry C*, 2010, 114(114):22181-22189.
2. Liu Y, Zhang B, Luo L, et al. TiO_2_/Cu_2_O Core/Ultrathin Shell Nanorods as Efficient and Stable Photocatalysts for Water Reduction. *Angewandte Chemie International Edition*, 2016, 54 (50): 15260-15265.
3. Bing M, Guan P Y, et al. MOF-derived Flower-like MoS_2_@TiO_2_ Nanohybrids with Enhanced Activity for Hydrogen Evolution. *ACS Appl Mater Interfaces*, 2016, 8 (40): 26794-26800.
4. He H, Lin J, Fu W, et al. MoS_2_/TiO_2_ Edge-On Heterostructure for Efficient Photocatalytic Hydrogen Evolution. *Advanced Energy Materials*, 2016, 6(14).
5. Li H, Wang Y, Chen G, et al. Few-layered MoS_2_ nanosheets wrapped ultrafine TiO_2_ nanobelts with enhanced photocatalytic property. *Nanoscale*, 2016, 8(11):6101-6109.
6. Hou H, Gao F, Wang L, et al. Superior thoroughly mesoporous ternary hybrid photocatalysts of TiO_2_/WO_3_/g-C_3_N_4_ nanofibers for visible-light-driven hydrogen evolution. *Journal of Materials Chemistry A*, 2016, 4(17):6276-6281.
7. Hou H, Shang M, Gao F, et al. Highly Efficient Photocatalytic Hydrogen Evolution in Ternary Hybrid TiO_2_/CuO/Cu Thoroughly Mesoporous Nanofibers. *ACS Applied Materials & Interfaces*, 2016, 8(31):20128.
8. Tamiolakis I, Papadas I T, Spyridopoulos K C, et al. Mesoporous assembled structures of Cu_2_O and TiO_2_ nanoparticles for highly efficient photocatalytic hydrogen generation from water. *RSC Advances*, 2016, 6(60):54848-54855.
9. Mondal I, Pal U. Synthesis of MOF templated Cu/CuO@TiO_2_ nanocomposites for synergistic hydrogen production. *PCCP*, 2016, 18(6):4780-4788.
10. Yue X, Yi S, Wang R, et al. Cobalt Phosphide Modified Titanium Oxide Nanophotocatalysts with Significantly Enhanced Photocatalytic Hydrogen Evolution from Water Splitting. *Small*, 2017, 13(14).
11. Marchal C, Cottineau T, Méndez-Medrano M G, et al. Au/TiO_2_-gC_3_N_4_ Nanocomposites for Enhanced Photocatalytic H_2_ Production from Water under Visible Light Irradiation with Very Low Quantities of Sacrificial Agents. *Advanced Energy Materials*, 2018:1702142.
12. Dörr T S, Deilmann L, Haselmann G, et al. Ordered Mesoporous TiO_2_ Gyroids: Effects of Pore Architecture and Nb-Doping on Photocatalytic Hydrogen Evolution under UV and Visible Irradiation. *Advanced Energy Materials*, 2018: 1802566.

Notably, we repeated the synthesis and photocatalyic test mentioned in reference 1 and 9, and got activity of 830 μmol/h and 175 μmol/h under the described conditions, respectively. There is a big difference between our data and the reported data, indicating further studies seem to be necessary to confirm the reproducibility.

**Supplementary Table 5.** Comparison of oxygen evolution reaction (OER) performance of different OER materials.

| Materials | Light source | Light intensity | Reactant solution | Activity  (μmol/h) | Reaction time | AQE | Ref. |
| --- | --- | --- | --- | --- | --- | --- | --- |
| LaTiO_2_N | λ>420nm | N/A | 0.05M AgNO_3_ | 736 | 2.5h | 27.1%  (440 nm) | 1 |
| Ta_3_N_5_@NaTaON | λ>400nm | 100 mW/cm^2^ | 0.8mM  Na_2_S_2_O_8_ | 546 | 0.5h | 16.8%  (420 nm) | 2 |
| CoO_x_-MgO-Ta_3_N_5_ | λ>420nm | N/A | 0.06M AgNO_3_ | 1200 | 0.5h | 11.3%  (500 nm) | 3 |
| CeO_2-x_S_x_  hollow spheres | UV-Vis(Xe) | N/A | 0.01M AgNO_3_ | 91 | 3h | N/A | 4 |
| S-g-C_3_N_4_/BiVO_4_ | λ>420nm | N/A | 0.05M AgNO_3_ | 75 | 6h | 8.32%  (488 nm) | 5 |
| NiCoP@NiCo–Pi/g-C_3_N_4_ | λ>420nm | N/A | 0.02M AgNO_3_ | 85.5 | 7h | 9.4%  (420 nm) | 6 |
| LiCuTa_3_O_9_ | λ>420nm | N/A | 0.01M AgNO_3_ | 19 | 1h | 0.21%  (420 nm) | 7 |
| Cd-TBAPy (MOF) | λ>420nm | N/A | 0.01M AgNO_3_ | 81.7 | N/A | 5.6%  (420 nm) | 8 |
| 3S-CFHoMS | UV-Vis(Xe) | 103  mW/cm^2^ | 0.01M AgNO_3_ | 452 | 5h | 6.8%  (365 nm) | This work |

1. Zhang F, Yamakata A, Maeda K, et al. Cobalt-modified porous single-crystalline LaTiO_2_N for highly efficient water oxidation under visible light. *Journal of the American Chemical Society*, 2012, 134(20):8348-51.

2. Hou J , Wu Y , Cao S , et al. In Situ Phase-Induced Spatial Charge Separation in Core-Shell Oxynitride Nanocube Heterojunctions Realizing Robust Solar Water Splitting. *Advanced Energy Materials*, 2017, 7(17):1700171.

3. Chen S, Shen S, Liu G, et al. Interface engineering of a CoO_(x)_/Ta_3_N_5_ photocatalyst for unprecedented water oxidation performance under visible-light-irradiation. *Angew. Chem. Int. Ed*. 2015, 54(10):3047-3051.

4. Xiao Y, Chen Y, Xie Y, et al. Hydrogenated CeO_2-x_S_x_ mesoporous hollow spheres for enhanced solar driven water oxidation. Chemical communications, 2016, 52(12):2521.

5. Kong H J, Da H W, Kim J, et al. Sulfur-Doped g-C_3_N_4_/BiVO_4_ Composite Photocatalyst for Water Oxidation under Visible Light. *Chemistry of Materials*, 2016, 28(5).

6. Qin Z, Chen Y, Huang Z, et al. A bifunctional NiCoP-based core/shell cocatalyst to promote separate photocatalytic hydrogen and oxygen generation over graphitic carbon nitride. *Journal of Materials Chemistry A*, 2017.

7. Dong B, Cui J, Liu L, et al. Development of Novel Perovskite-Like Oxide Photocatalyst LiCuTa_3_O_9_ with Dual Functions of Water Reduction and Oxidation under Visible Light Irradiation. *Adv. Energy Mater.* 2018, 8, 1801660.

8. Yejun X, Yu Q, Xiuli W, et al. Visible-Light-Responsive 2D Cadmium-Organic Framework Single Crystals with Dual Functions of Water Reduction and Oxidation. *Advanced Materials*, 2018, 1803401.

**Supplementary Table 6.** The comparison of overall water splitting performance of photocatalysts in other works.

| Catalyst | Solution | Cocatalyst | Light Source | HER Rate  (umol/h) | OER Rate  (umol/h) | Ref. |
| --- | --- | --- | --- | --- | --- | --- |
| BaTaO_2_N/WO_3_ | NaI (1 mM) | 3 wt% Pt | λ>420 nm(Xe) | 3.2 | 1.6 | 1 |
| La5Ti2Cu0.9Ag0.1S5O7/Au/BiVO_4_ | Pure water | Rh/Cr_2_O_3_ | λ>420 nm(Xe) | 22 | 11 | 2 |
| C-Dots/C_3_N_4_ | Pure water | N/A | λ>420 nm(Xe) | 8.4 | 4.1 | 3 |
| SrTiO_3_:Rh/  BiVO_4_ | Pure water | 1 wt% Ru | λ>400 nm(Xe) | 17.5 | 8.1 | 4 |
| SrTiO_3_:La,Rh/  Au/BiVO_4_:Mo | Pure water | Ru/RuO_x_ | AM 1.5G | 100 | 50 | 5 |
| Cring-g-C_3_N_4_ | Pure water | 3 wt% Pt | UV-Vis(Xe) | 11 | 5.5 | 6 |
| TCHoMS/  CFHoMS | FeCl_3_ (2 mM) | 1 wt% Pt/  3 wt% Ru | UV-Vis(Xe) | 9.8 | 4.6 | This work |
| TCHoMS/  CFHoMS | FeCl_3_ (2 mM) | 1 wt% Pt/  3 wt% Ru | AM 1.5G | 1.5 | 0.7 | This work |

1. Qi Y, Chen S, Li M, et al. Achievement of visible-light-driven Z-scheme overall water splitting using barium-modified Ta_3_N_5_ as a H_2_-evolving photocatalyst. Chemical Science, 2016, 8(1):437.
2. Sun S, Hisatomi T, Wang Q, et al. Efficient Redox-Mediator-Free Z-Scheme Water Splitting Employing Oxysulfide Photocatalysts under Visible Light. ACS Catalysis, 2018, 8(3):1690.
3. Liu J, Liu Y, Liu N, et al. Metal-free efficient photocatalyst for stable visible water splitting via a two-electron pathway. Science, 2015, 46(23):970-974.
4. Jr M A M, Wu Z, Nail B A, et al. Surface Photovoltage Measurements on a Particle Tandem Photocatalyst for Overall Water Splitting. Nano Letters, 2017, 18(2).
5. Wang Q, Hisatomi T, Jia Q, et al. Scalable water splitting on particulate photocatalyst sheets with a solar-to-hydrogen energy conversion efficiency exceeding 1%. Nature Materials, 2016, 15(6):611.
6. Che W, Cheng W, Yao T, et al. Fast Photoelectron Transfer in (Cring)-C_3_N_4_ Plane Heterostructural Nanosheets for Overall Water Splitting. Journal of the American Chemical Society, 2017, 139(8):3021.
